# Camel milk is associated with ameliorated high-fat diet-induced obesity and metabolic disorders via modulation of gut microbiota and remodeling of hepatic lipid metabolism: a multi-omics analysis

**DOI:** 10.3389/fnut.2026.1913436

**Published:** 2026-10-22

**Authors:** Bin Yang, Jingjing Wang, Weijie Pan, Hao Liang, Hongxia Zhao

**Affiliations:** Inner Mongolia Key Laboratory of Veterinary Fundamentals and Disease Control of Herbivorous Livestock, College of Veterinary Medicine, Inner Mongolia Agricultural University, Hohhot, China

**Keywords:** camel milk, gut microbiota, gut-liver axis, high-fat diet, lipid metabolism, obesity

## Abstract

This study investigated the therapeutic effects of camel milk (CM) on high-fat diet (HFD)-induced obesity and its underlying mechanisms through integrative multi-omics analysis. C57BL/6J mice were fed a normal diet (NC), an HFD, or an HFD supplemented with CM via intragastric administration for 7 weeks, with orlistat as a positive control. CM treatment significantly reduced body weight, inguinal and epididymal white adipose tissue weights, serum triglyceride and LDL-C levels, and adipocyte hypertrophy compared to the HFD group. Additionally, CM ameliorated HFD-induced hepatic injury, as evidenced by reduced serum ALT levels and attenuated hepatic steatosis. 16S rRNA gene sequencing revealed that CM partially reversed HFD-induced gut microbiota dysbiosis, characterized by increased Bacteroidota abundance, suppression of pro-inflammatory genera (*Mucispirillum, Escherichia-Shigella, Faecalitalea*), and enrichment of the putative beneficial taxon *unclassified [Eubacterium]_coprostanoligenes_group*. Targeted metabolomics revealed that CM restored HFD-suppressed levels of colonic short-chain fatty acids (SCFAs), including acetic, butyric, and propionic acids. Untargeted liver metabolomics revealed that CM remodeled the hepatic metabolome, reversing HFD-induced elevations in sphingolipids [sphingosine, SM(d18:0/16:1(9Z))], and modulating lipid, glutathione metabolisms, and oxidative stress pathway. Liver transcriptomic analysis indicated that CM partially reversed HFD-induced gene expression changes, particularly downregulating genes involved in arachidonic acid metabolism (Cyp4a family), fatty acid elongation (Acot family), and glycerolipid metabolism (*Aldh3a2, Mgll, Mogat1*), while modulating bile secretion genes. Spearman correlation analyses further revealed significant associations between CM-modulated gut microbial genera (notably *unclassified [Eubacterium]_coprostanoligenes_group, Faecalitalea*, and *Escherichia-Shigella*) and obesity-related parameters, SCFA levels, hepatic metabolites, and lipid metabolism-related gene expression. These findings suggest that CM attenuates HFD-induced obesity and associated metabolic disturbances in association with gut microbiota modulation, enhanced SCFA production, and remodeling of hepatic lipid metabolism involving the gut-liver axis, pointing to CM as a potential dietary intervention for obesity management.

## 1 Introduction

Obesity is a global epidemic, and in 2021, about 2.11 billion adults were classified as overweight or obese, projected to reach 3.8 billion by 2050. Rising global childhood obesity suggests this trend will continue, escalating the burden of non-communicable diseases such as diabetes, heart disease, and cancer. Effective interventions are urgently needed to avert the foreseeable rise in obesity-related disease burdens (1). Management strategies for obesity range from lifestyle interventions (e.g., diet, physical activity, and behavioral therapy) to pharmacological treatment. Common anti-obesity medications pose significant risks, from organ damage to teratogenicity. However, their contraindications and potential adverse effects must be carefully considered in clinical management (2). The limitations and adverse effects associated with current therapeutic regimens have spurred considerable scientific interest in natural bioactive compounds from food, which are regarded as safer and more effective alternatives with significant anti-obesity potential (3).

Camel milk (CM), traditionally consumed in many regions as a food rich in nutrients (such as insulin-like proteins, lactoferrin, and unsaturated fatty acids) and bioactive components (like bioactive peptides and extracellular vesicles), has been reported in recent studies to exhibit potential in anti-diabetic, anti-obesity, and anti-inflammation (4–6). However, its precise molecular mechanisms remain unclear, which limits its development and promotion as a scientifically endorsed health food.

Despite hypotheses that CM confers benefits for conditions such as inflammatory bowel disease, mental disorders, allergies, and obesity by modulating the gut microbiota, direct evidence for its anti-obesity effects and its underlying mechanisms is lacking (4). The pathways through which it systemically regulates host metabolism and the essential multi-organ crosstalk involved remain elusive.

In recent years, the “gut-liver axis” has been recognized as a key hub regulating energy metabolism and immune homeostasis. A high-fat diet can disrupt the balance of the gut microbiota, leading to reduced beneficial metabolites such as short-chain fatty acids (SCFAs) and to impairment of the intestinal barrier, which, in turn, affects hepatic metabolism via the portal vein (7). We propose that the beneficial effects of CM may be mediated through the modulation of this axis.

To verify this hypothesis and comprehensively elucidate the underlying mechanisms, this study utilized a multi-omics integration strategy. Based on the evaluation of the ameliorative effects of CM gavage on physiological and biochemical indicators in a diet-induced obese mouse model, we simultaneously analyzed changes in gut microbiota structure, colonic SCFA levels, and liver transcriptomic and metabolomic profiles. By integrating these multidimensional datasets, we aimed to construct a holistic network connecting gut microbial ecology with hepatic metabolism and gene regulation. This work is anticipated to provide novel mechanistic insights into the health-promoting effects of CM, thereby reinforcing its scientific standing as a functional food.

## 2 Materials and methods

### 2.1 Animals and experimental design

CM was collected during the mid-lactation period from Ejina Banner, Inner Mongolia. Fresh camel milk was collected and immediately placed in 50 mL centrifuge tubes. The samples were transported to the laboratory on ice, with the cold chain maintained for approximately 4 h. Upon arrival, the milk was aliquoted into 5 mL centrifuge tubes and stored at −80 °C. The storage duration at −0 °C did not exceed 3 months. The milk was raw and not pasteurized. Before gavage, gavage, the frozen aliquots were thawed in a 37 °C water bath and administered immediately. Aliquoting into single-use volumes ensured that no repeated freeze-thaw cycles occurred. The nutritional composition of camel milk, including fat, protein, lactose, and total solids, was determined using a MilkoScan 7 RM (Foss, Denmark). The data are presented in Supplementary Table S1. Five-week-old male C57BL/6J mice were purchased from SPF Biotechnology Co., Ltd. (Beijing, China). All animal experiments were approved by the Experimental Animal Welfare and Ethics Committee of Inner Mongolia Agricultural University (Approval No. NND2025037). The experimental timeline diagram illustrates the sequence and duration of each experimental phase (Supplementary Figure S1). The mice were housed under controlled conditions at 22 °C ± 1 °C, 50% ± 5% relative humidity, and a 12 h light/dark cycle, and were provided with food and water *ad libitum*. Mice were randomly assigned to experimental groups after 1 week of acclimatization. Randomization was performed using a computer-generated random number sequence, with stratification by body weight to ensure comparable initial body weights across groups. The mice were randomly assigned to four groups (*n* = 8 per group, with 2 mice per cage). One group served as the normal control (NC) and was fed a standard diet (F-003, purchased from SPF Biotechnology Co., Ltd., China). The remaining three groups were designated as high-fat diet (HFD) groups and were fed a high-fat diet (D12492, purchased from SPF Biotechnology Co., Ltd., China). After 10 weeks of dietary intervention, the NC group and one HFD group were orally administered 10 mL/kg body weight (BW) of saline (HFD group), while the other two HFD groups received either 40 mg/kg BW of orlistat (orlistat group) or 10 mL/kg BW of camel milk (CM group) daily by gavage. The CM dose of 10 mL/kg/day used in this study was selected based on previously published studies in which oral gavage of camel milk at this dose demonstrated significant biological effects in rodent models (8, 9). The treatments were administered once daily for 7 weeks. BW and food intake were measured weekly. At the end of the feeding period, all mice were fasted for 12 h and euthanized. BW was measured immediately before euthanasia. Mice were anesthetized with isoflurane inhalation, after which retro-orbital blood collection was performed. Subsequently, the mice were euthanized by cervical dislocation. Following euthanasia, the abdominal skin was incised, and the inguinal white adipose tissue (iWAT) was dissected, weighed, and immediately placed in 10% formalin fixative. The abdominal cavity was then opened, and the epididymal white adipose tissue (eWAT) was removed, weighed, and fixed in 10% formalin. The liver was excised and weighed, and then divided into four portions: one portion was fixed in 10% formalin for hematoxylin and eosin (HE) staining, one portion was fixed in 4% paraformaldehyde for Oil Red O staining, one portion was snap-frozen in liquid nitrogen for untargeted hepatic metabolomic analysis, and the remaining portion was snap-frozen in liquid nitrogen for hepatic transcriptomic analysis. Colonic contents were collected aseptically and divided into two aliquots: one was snap-frozen in liquid nitrogen for gut microbiota analysis and the other was snap-frozen in liquid nitrogen for short-chain fatty acid (SCFA) analysis. All samples stored in liquid nitrogen were subsequently transferred to −80 °C. All procedures were carried out on ice to preserve biomolecule integrity.

### 2.2 Random blood glucose (RBG), fasting blood glucose (FBG), oral glucose tolerance test (OGTT) and insulin tolerance test (ITT)

Blood glucose measurements were performed using a glucometer manufactured by Roche Diabetes Care GmbH, model Accu-Chek Active, serial number GB22275505. RBG levels were measured in non-fasted mice using a portable glucometer at week 17. OGTT was performed at week 17. Mice were fasted for 12 h and then administered a glucose solution (2 g/kg BW) by oral gavage. Blood glucose levels were measured from the tail vein immediately before (0 min) and at 30, 60, 90, and 120 min after glucose administration using a portable glucometer. The area under the curve (AUC) for the OGTT was calculated using the trapezoidal rule. ITT was performed at week 17. Mice were fasted for 6 h and then injected intraperitoneally with insulin (1 U/kg BW). Blood glucose levels were measured from the tail vein immediately before (0 min) and at 30, 60, 90, and 120 min after insulin injection using a portable glucometer. The AUC for the ITT was calculated using the trapezoidal rule. FBG was measured at week 18. After a 12 h fast, blood was collected from the tail vein of mice, and FBG was determined using a portable glucometer.

### 2.3 Biochemical analysis

Following blood collection at the end of the experiment, serum was separated by centrifugation at 2,000 × g for 15 min and stored at −80 °C for subsequent use. Fasting serum insulin (FINS) was measured using a commercial ELISA kit (Mmbio, Jiangsu, China). Insulin resistance was evaluated using the homeostasis model assessment (HOMA-IR). The index was calculated according to the following formula: HOMA-IR = (FBG × FINS) / 22.5 (FBG: mmol/L; FINS: mIU/L).

Serum levels of triglycerides (TG), total cholesterol (TC), low-density lipoprotein cholesterol (LDL-C), high-density lipoprotein cholesterol (HDL-C), as well as the liver enzymes alanine aminotransferase (ALT) and aspartate aminotransferase (AST), were measured using commercial assay kits (Nanjing Jiancheng Bioengineering Institute, Jiangsu, China) according to the manufacturers' instructions.

### 2.4 Histopathological examination

Liver, epididymal white adipose tissue, and inguinal white adipose tissue samples were fixed in 10% formalin. After fixation, tissues were trimmed and placed in labeled embedding cassettes, then rinsed in running tap water for 12 h to remove residual fixative. The samples were dehydrated through a graded ethanol series (75%, 85%, 95%, and absolute ethanol), each step lasting 15–30 min, cleared in xylene, and subsequently infiltrated and embedded in paraffin. Paraffin blocks were sectioned at a thickness of 5 μm using a rotary microtome. Sections were flattened in a 37 °C water bath, collected on adhesive slides, and baked on a 40 °C hot plate for 1 h.

For staining, the sections were deparaffinized in xylene (three changes, 5 min each) and rehydrated through a descending ethanol series (absolute ethanol twice, 2 min each; 95%, 85%, and 75% ethanol, 1 min each). The sections were then stained with hematoxylin for 10 min and rinsed in tap water to remove excess stain. Differentiation was performed in 1% hydrochloric acid ethanol for 15 s, followed by tap water rinsing. Bluing was carried out in 1% sodium bicarbonate for 15 s, followed by tap water rinsing. Counterstaining was performed with 1% eosin for 1 min, and the sections were again rinsed in tap water. After staining, the sections were dehydrated through an ascending ethanol series (85% ethanol for 5 s; 95% ethanol twice, 10 s each; absolute ethanol twice, 5 min each), cleared in xylene (three changes, 5 min each), and briefly air-dried. The sections were mounted with neutral balsam and baked at 50 °C for 3 h. Once completely dry, the slides were examined and images were captured using a pathological slide scanner.

For hepatic lipid analysis, liver tissues were cryoprotected overnight in 30% sucrose, embedded in OCT medium, and serially sectioned at a thickness of 10 μm. The frozen sections were then stained with Oil Red O (ORO) to visualize neutral lipids.

### 2.5 16S rRNA gene sequencing and analysis of the gut microbiome

Colonic contents from the NC, HFD, and CM groups (each with six biological replicates) were collected for gut microbiome analysis. Genomic DNA was extracted from all samples using the cetyltrimethylammonium bromide (CTAB) method, and its integrity was verified by electrophoresis on 1% agarose gels. The V4 hypervariable region of the bacterial 16S rRNA gene was amplified with barcode-indexed primers (515F-06R). Polymerase chain reaction (PCR) was performed using Phusion^®^ High-Fidelity PCR Master Mix (New England Biolabs). The resulting amplicons were separated on 2% agarose gels and purified with a Qiagen Gel Extraction Kit (Qiagen, Germany). Sequencing libraries were prepared with appropriate indices, followed by quality assessment using Qubit fluorometry, quantitative real-time PCR, and Bioanalyzer-based size distribution analysis. Libraries were pooled based on their effective concentrations and the required sequencing depth, and were subsequently sequenced on the Illumina NovaSeq 6000 platform.

Bioinformatic analysis of the sequencing data was subsequently performed. Paired-end reads were demultiplexed based on unique barcodes, and adapter/primer sequences were trimmed. Reads were merged using FLASH (v1.2.11) to generate raw tags. Quality control was applied using the QIIME (v1.9.1) pipeline, with specific filtering parameters, to produce high-quality, clean tags. The clean tags were clustered into operational taxonomic units (OTUs) at a 97% similarity threshold using Uparse (v7.0.1001). Taxonomic annotation was performed against the Silva 138.1 database. Multiple sequence alignment was conducted with MUSCLE (v3.8.31). To enable comparative analysis, OTU abundance data were normalized by subsampling to the minimum number of sequences per sample. The resulting normalized OTU table was used for all subsequent analyses.

To evaluate between-sample (beta) diversity and community structure, principal coordinate analysis (PCoA) and non-metric multidimensional scaling (NMDS) were performed in R using a Bray-Curtis dissimilarity matrix. Furthermore, linear discriminant analysis effect size (LEfSe) was applied to identify specific bacterial taxa that were significantly enriched, with a linear discriminant analysis (LDA) score cutoff of >4.

### 2.6 Analysis of short-chain fatty acids (SCFAs) in colonic contents

Colonic contents from the NC, HFD, and CM groups (each with three biological replicates) were collected for targeted metabolomic analysis. The samples were resuspended using liquid nitrogen and subsequently homogenized with 80% methanol, followed by centrifugation at 12,000 rpm for 10 min to remove proteins. The resulting supernatant was mixed with 150 μL of derivatization reagent and incubated at 40 °C for 40 min. After derivatization, the sample was diluted with 80% methanol. Then, 95 μL of the supernatant was combined with 5 μL of a mixed internal standard solution and thoroughly homogenized. Finally, the mixture was injected into the LC-MS/MS system for analysis. An ultra-high-performance liquid chromatography-tandem mass spectrometry (UHPLC-MS/MS) system (Vanquish™ Flex UHPLC-TSQ Altis™, Thermo Scientific, Germany) was used to quantify short-chain fatty acids (SCFAs). The analysis was performed at Novogene Co., Ltd. (Beijing, China). The SCFAs measured included acetic acid, propionic acid, isobutyric acid, butyric acid, 2-methylbutyrate, isovaleric acid, valeric acid, and hexanoic acid. The detailed methods were shown in the supplementary methods file.

### 2.7 Untargeted liver metabolomics by UHPLC–MS/MS

Liver tissues from the NC, HFD, and CM groups (with six biological replicates per group) were prepared for untargeted metabolomic analysis. untargeted metabolomic analysis of liver tissue was conducted via ultra-high performance liquid chromatography-tandem mass spectrometry (UHPLC–MS/MS). Metabolites were extracted from 100 mg of liver tissue and analyzed by LC–MS.

UHPLC-MS/MS analysis was carried out on a Vanquish UHPLC system (Thermo Fisher) coupled to an Orbitrap Q Exactive™ HF mass spectrometer (Thermo Fisher, Germany) at Novogene Co., Ltd. Separation was achieved using an ACQUITY UPLC BEH amide column (100 × 2.1 mm, 1.7 μm). The detailed methods were shown in the Supplementary material.

Metabolite annotation was performed using the KEGG (https://www.genome.jp/kegg/pathway.html), HMDB (https://hmdb.ca/metabolites), and LIPIDMaps (http://www.lipidmaps.org/) databases. Principal component analysis (PCA) was conducted with the metaX software. Univariate analysis (*t*-test) was used to determine statistical significance (*p*-value). Differential metabolites were identified based on the following criteria: variable importance in projection (VIP) > 1.0, fold change (FC) > 1.2 or FC < 0.833, and *p* < 0.05.

### 2.8 RNA sequencing and transcriptomic data analysis

Liver tissues from the NC, HFD, and CM groups (each with 3 biological replicates) were collected for total RNA extraction. Total RNA was isolated with TRIzol reagent (Invitrogen), followed by treatment with RNase-free DNase I (Takara, Dalian, China) to eliminate genomic DNA contamination. mRNA purity, concentration, and integrity were assessed using a NanoDrop spectrophotometer, Qubit fluorometer, and Agilent 2100 Bioanalyzer, respectively. Poly(A)+ mRNA was subsequently enriched with Dynabeads Oligo(dT) (Invitrogen) and fragmented in fragmentation buffer. First-strand cDNA synthesis was performed using random hexamer primers and SuperScript II reverse transcriptase (Invitrogen), after which double-stranded cDNA was generated and purified with AMPure XP beads. The resulting cDNA was amplified by PCR to construct sequencing libraries, which were finally subjected to paired-end sequencing on an Illumina HiSeq platform at Novogene.

Raw sequencing reads were processed with fastp software to remove adapter sequences and low-quality bases. Clean paired-end reads were then aligned to the mouse reference genome using Hisat2 (version 2.2.1). Gene-level read counts were quantified with FeatureCounts (version 2.0.6). The expression level of each gene was calculated as FPKM (fragments per kilobase of transcript per million mapped reads) based on its length and the corresponding mapped read count. Differential expression analysis between groups was conducted using the DESeq2 R package (version 1.42.0) on the raw gene-level read counts, which employs a negative binomial model to assess statistical significance in digital gene expression data. Resulting *p*-values were adjusted for multiple testing using the Benjamini–Hochberg procedure to control the false discovery rate. Genes meeting the criteria of adjusted *p* < 0.05 and |log_2_ (fold change)| ≥ 1 were identified as differentially expressed. Subsequently, KEGG pathway enrichment analysis was performed on the differentially expressed gene sets using the clusterProfiler R package, with reference to KEGG pathway annotations.

### 2.9 Blinding

Blinding was implemented during data collection and analysis. Specifically, all biological samples and sequencing data were coded by a researcher not involved in the downstream analyses. The investigators performing biochemical assays, sequencing library preparation, and bioinformatics analyses were blinded to group allocation.

### 2.10 Statistical analysis

For multiple-group comparisons, one-way analysis of variance (ANOVA) or two-way repeated-measures ANOVA was performed, followed by Tukey's *post hoc* test to adjust for multiple comparisons. When the assumptions of normality was violated, the non-parametric Kruskal–Wallis test with Dunn's multiple comparison test was used. Statistical analyses of correlation were performed using the Spearman correlation test in IBM SPSS Statistics 19 (IBM Corp., Armonk, NY, USA). The data were presented as mean ± standard deviation (SD). A two-tailed *p* value < 0.05 was considered statistically significant.

## 3 Result

### 3.1 Effects of CM on BW, food intake, fat accumulation, serum lipids, and adipocyte morphology in HFD-induced obese mice

To investigate the therapeutic potential of CM against HFD-induced obesity, a 17-week study was conducted in C57BL/6J mice. The animals were assigned to one of four groups: a normal diet control group (NC), an HFD control group, an HFD group receiving daily CM via intragastric gavage, and an HFD group treated with orlistat as a positive control. BW, food intake, inguinal and epididymal fat mass, and serum lipid profiles were assessed. Compared with the NC group, the BW of mice in the HFD group significantly increased from week 10 to week 17 (*p* < 0.01; Figure 1A). Food intake significantly decreased at weeks 11 and 17 (*p* < 0.05), but showed no significant difference from week 12 to week 16 (*p* > 0.05; Figure 1B). The weights of iWAT and eWAT were significantly increased (*p* < 0.05; Figure 1C). Serum TG levels increased but not significantly (*p* > 0.05), while TC and LDL-C levels were significantly elevated (*p* < 0.01) (Figure 1D). Notably, compared with the HFD group, the CM group exhibited a significant reduction in BW at weeks 13, 14, 15, and 16 (*p* < 0.01; Figure 1A) (Supplementary Table S3). Food intake was significantly decreased at weeks 14 and 15 (*p* < 0.05; Figure 1B) (Supplementary Table S4). The weights of iWAT and eWAT were significantly decreased (*p* < 0.01; Figure 1C). Furthermore, TG (*p* < 0.01) and LDL-C (*p* < 0.05) were notably reduced, while HDL-C levels were significantly elevated (*p* < 0.01; Figure 1D). Compared with the HFD group, The orlistat group showed no weight loss across the weeks (*p* > 0.05) (Figure 1A). No significant change in food intake was observed from week 11 to week 16 (*p* > 0.05). It was significantly elevated at week 17 (*p* < 0.05) (Figure 1B). The weights of iWAT and eWAT were significantly decreased (*p* < 0.01; Figure 1C). Additionally, LDL-C levels were significantly reduced (*p* < 0.05), while HDL-C levels were significantly increased (*p* < 0.01; Figure 1D). Results of H&E staining revealed a reduction in adipocyte area in both iWAT and eWAT from mice treated with CM or orlistat (Figure 1E).

**Figure 1:**
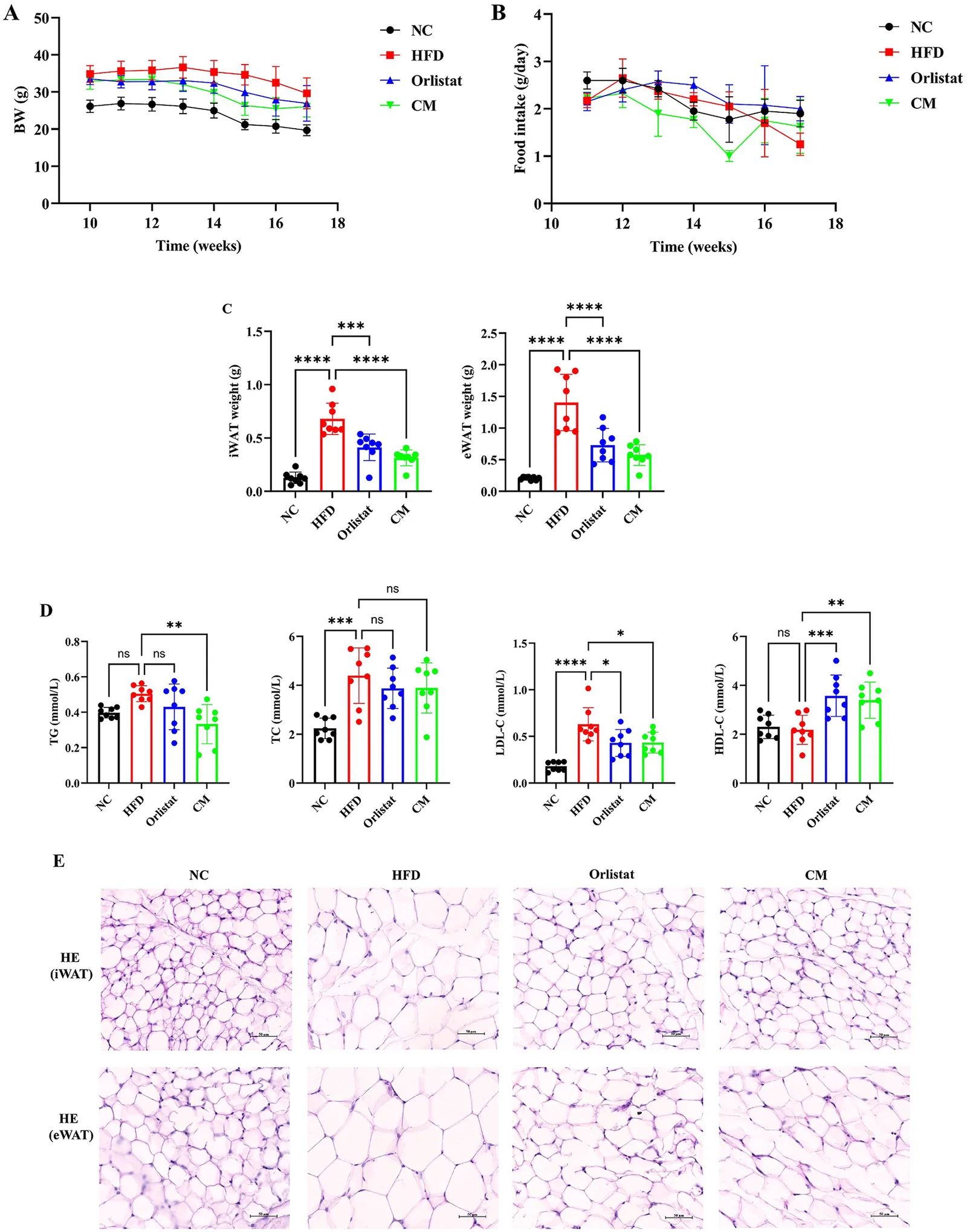
CM attenuated HFD-induced obesity and hyperlipidemia in mice. **(A)** Changes in the BW of mice treated with CM for 8 weeks. **(B)** Changes in food intake of mice treated with CM for 7 weeks. **(C)** Weight of iWAT and eWAT at week 18. **(D)** Serum lipid levels (TC, TG, LDL-C, HDL-C). **(E)** H&E staining of iWAT and eWAT. Data are expressed as mean ± standard deviation (*n* = 8). Significance was determined by two-way repeated-measures ANOVA or one-way ANOVA with Tukey's *post hoc* test. *p < 0.05, **p < 0.01, ***p < 0.001, ****p < 0.0001. ns, not significant.

### 3.2 Effects of CM treatment on glycemic control, insulin sensitivity, and hepatic steatosis in HFD-induced obese mice

After 17 weeks of treatment, no significant differences in FBG levels were observed among the four groups (*p* > 0.05). The RBG levels in the HFD-fed mice were higher than those in the NC group, but not significantly (*p* > 0.05). Compared with the HFD group, the orlistat group showed a significant reduction in RBG levels (*p* < 0.05), while the CM group showed a non-significant decrease (*p* > 0.05) (Figure 2A). No significant differences were observed in serum insulin levels or HOMA-IR among the four groups of mice (*p* > 0.05; Figure 2B).

**Figure 2:**
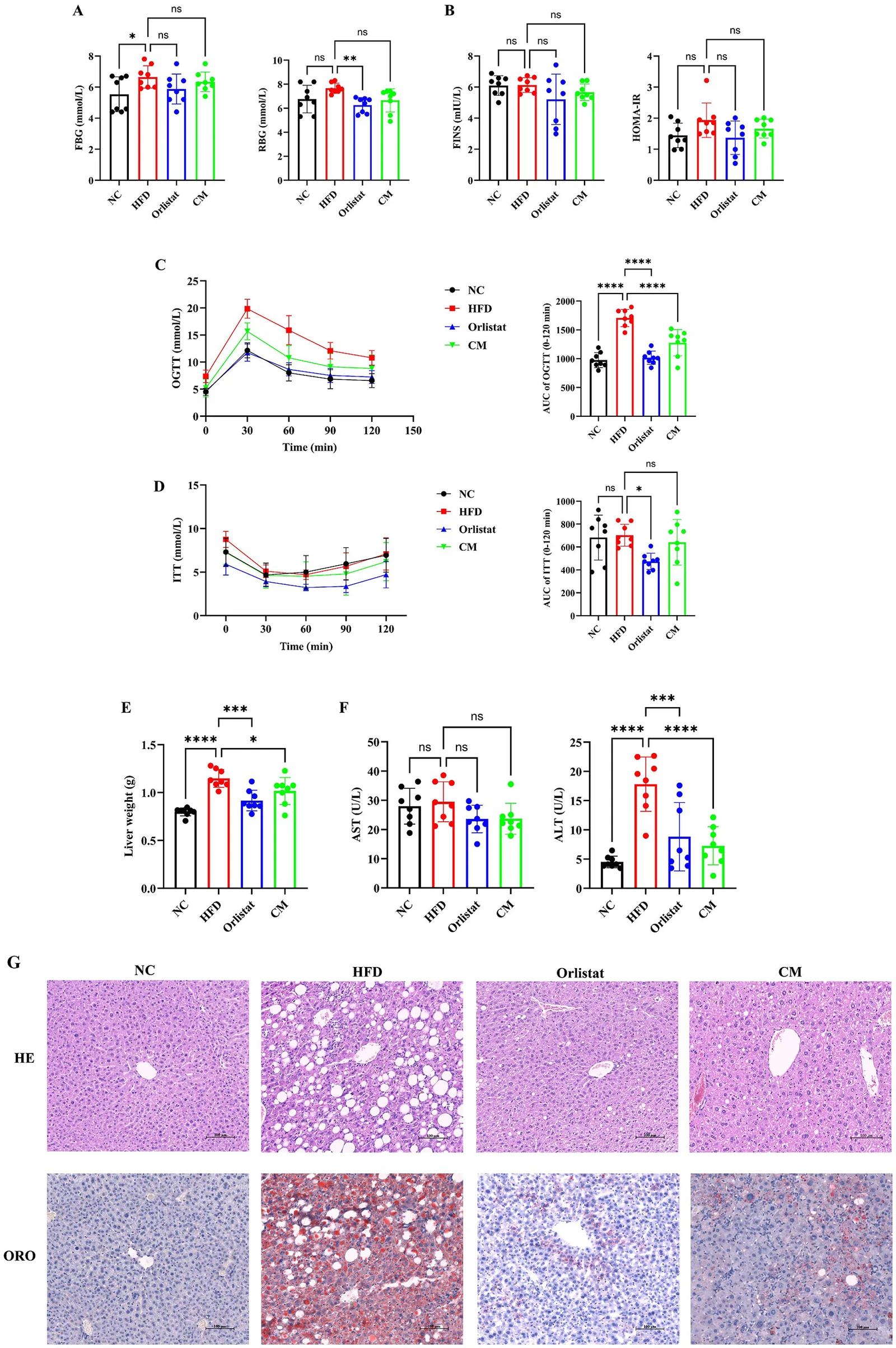
CM improved glucose metabolism and reduced hepatic lipid deposition in HFD-induced obese mice. **(A)** The FBG and RBG levels. **(B)** Fasting serum insulin and HOMA-IR. **(C)** Changes in blood glucose and AUC during OGTT. **(D)** Changes in blood glucose and AUC during ITT. **(E)** Liver weight at week 8. **(F)** Serum AST and ALT levels. **(G)** H&E and ORO staining of liver. Data are expressed as mean ± standard deviation (*n* = 8). Significance was determined by two-way repeated-measures ANOVA or one-way ANOVA with Tukey's *post hoc* test. *p < 0.05, **p < 0.01, ***p < 0.001, ****p < 0.0001. ns, not significant.

To evaluate glucose metabolism, an OGTT was performed in mice after intragastric glucose administration. The OGTT results showed that the HFD group maintained the highest blood glucose levels throughout the test, indicating impaired glucose tolerance. Both the orlistat and CM treatment groups exhibited improved glycemic control compared to the HFD group, with orlistat demonstrating a more pronounced glucose-lowering effect, closely resembling the NC group response (Figure 2C) (Supplementary Table S5). To assess insulin resistance, an ITT was performed following intraperitoneal insulin injection. The results showed that insulin sensitivity was significantly improved in the orlistat-treated group (*p* < 0.05) compared to the HFD group. A non-significant improvement was observed in the CM group (*p* > 0.05; Figure 2D) (Supplementary Table S6).

To assess hepatic injury and steatosis, liver weight, serum AST and ALT levels, and histopathological alterations were evaluated in mice from each group. The results indicated that the HFD group had a significantly higher liver weight than the NC group (*p* < 0.05). Compared with the HFD group, orlistat-treated mice showed a significant reduction in liver weight (*p* < 0.01). In contrast, the CM group demonstrated a non-significant decrease (*p* > 0.05; Figure 2E). There were no significant differences in serum AST levels among the groups (*p* > 0.05). Serum ALT levels were significantly higher in the HFD group compared to the NC group (*p* < 0.01). Compared with the HFD group, both the orlistat-treated and CM groups showed significant reductions in serum ALT levels (*p* < 0.01; Figure 2F). Hepatic steatosis accompanied by lipid droplet formation was observed in HFD-fed mice compared to the NC group. These changes were significantly attenuated in both the orlistat-treated and CM groups (Figure 2G).

### 3.3 Alteration of gut microbiota by CM in HFD-fed mice

PCoA of gut microbiota β-diversity showed separation between the NC and HFD groups along PC1 (68.43% variance). The CM-treated group exhibited an intermediate distribution between NC and HFD clusters along PC2 (7.67% variance), suggesting partial modulation of HFD-induced microbial shifts (Figure 3A). NMDS analysis (Stress = 0.036) revealed distinct separation between the NC and HFD groups. The CM group clustered intermediately, indicating that CM gavage partially restored the HFD-induced shifts in gut microbiota composition (Supplementary Figure S2A).

**Figure 3:**
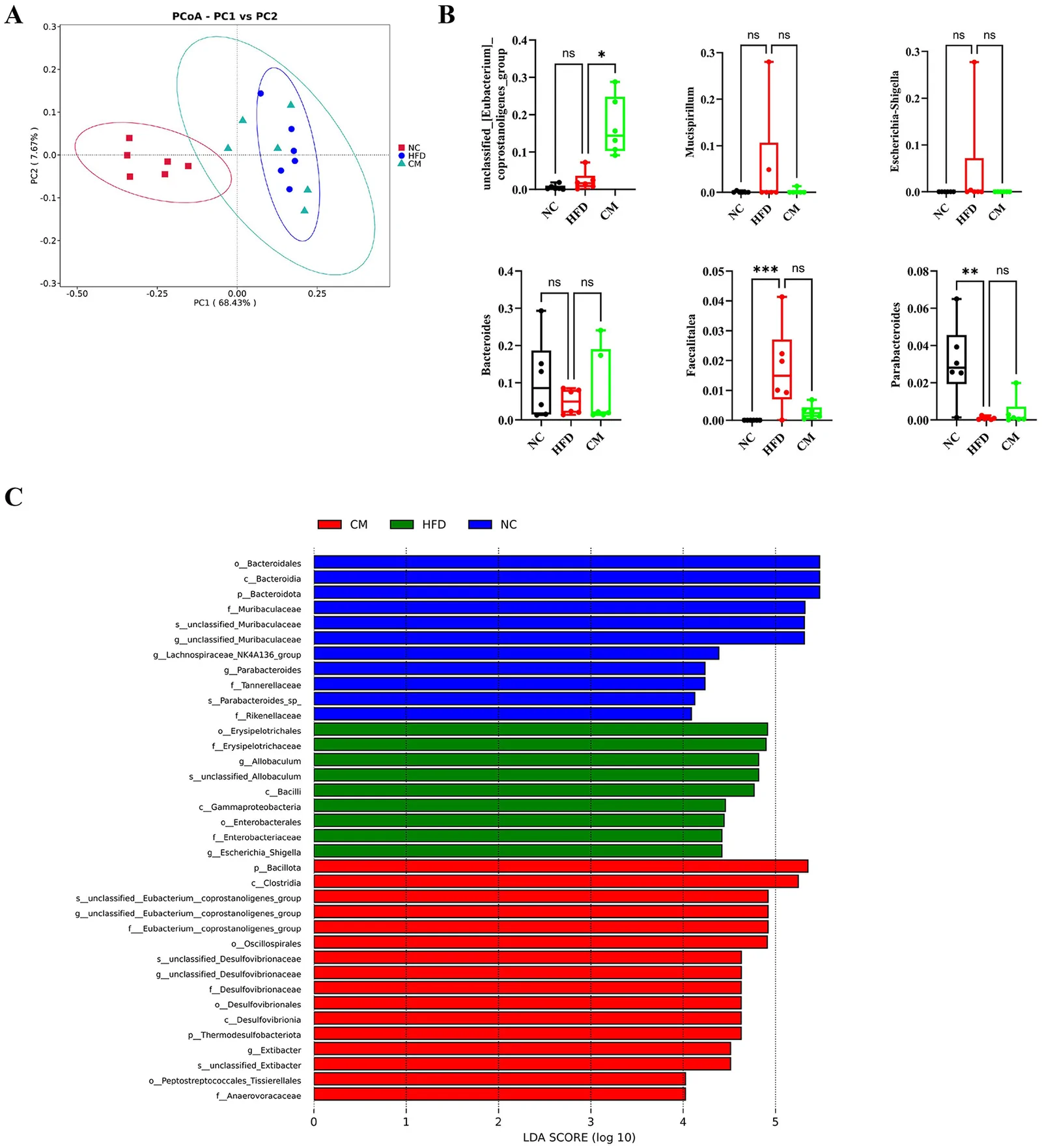
CM altered the gut microbiota composition in HFD-induced obese mice. **(A)** PCoA of gut microbiota. **(B)** Variation in microbiota at the genus level. **(C)** LEfSe analysis. Data are expressed as mean ± standard deviation (*n* = 6). Significance was determined by Kruskal–Wallis test with Dunn's multiple comparison test. *p < 0.05, **p < 0.01, ***p < 0.001. ns, not significant.

At the phylum level, compared with normal chow-fed mice, HFD feeding significantly reduced the abundance of Bacteroidota and increased the levels of Pseudomonadota and Deferribacterota. Treatment with CM increased Bacteroidota and markedly decreased Pseudomonadota and Deferribacterota, shifting their relative abundances closer to those observed in the NC group. These findings suggest that CM modulates HFD-induced gut microbiota dysbiosis (Supplementary Figure S2B). CM administration modulated HFD-induced gut dysbiosis at the genus level. The HFD group showed a marked increase in the relative abundance of *Mucispirillum, Escherichia-Shigella*, and *Faecalitalea*, along with a decrease in *Bacteroides* and *Parabacteroides*, compared to the NC group. CM treatment effectively reversed these changes by significantly suppressing the HFD-enriched genera *Mucispirillum, Escherichia-Shigella*, and *Faecalitalea* and partially restoring the abundance of *Bacteroides*. Notably, the *unclassified [Eubacterium]_coprostanoligenes_group* was substantially elevated in the CM group (Supplementary Figure S2C and Figure 3B).

LEfSe analysis identified differentially abundant microbial taxa across the NC, HFD, and CM groups. The HFD group showed an increase in pro-inflammatory taxa (*Escherichia-Shigella*). CM treatment significantly modulated this profile, enriching for potential beneficial taxa including members of the *Desulfovibrionaceae* family and the *Eubacterium_coprostanoligenes_group* (Figure 3C). This shift suggests that CM mitigates HFD-induced gut microbial dysbiosis.

### 3.4 Alterations in colonic SCFA concentrations following CM intervention

The concentrations of short-chain fatty acids (SCFAs) in colonic contents were measured across the three experimental groups. Compared to the NC group, the HFD group exhibited lower concentrations of acetic acid, butyric acid, isovaleric acid, valeric acid, hexanoic acid, and propionic acid. Conversely, CM treatment elevated the levels of acetic acid, isobutyric acid, butyric acid, 2-methylbutyrate, isovaleric acid, valeric acid, hexanoic acid, and propionic acid compared to the HFD group. In the CM group, the levels of acetic acid and propionic acid were comparable to those in the NC group (Figure 4).

**Figure 4:**
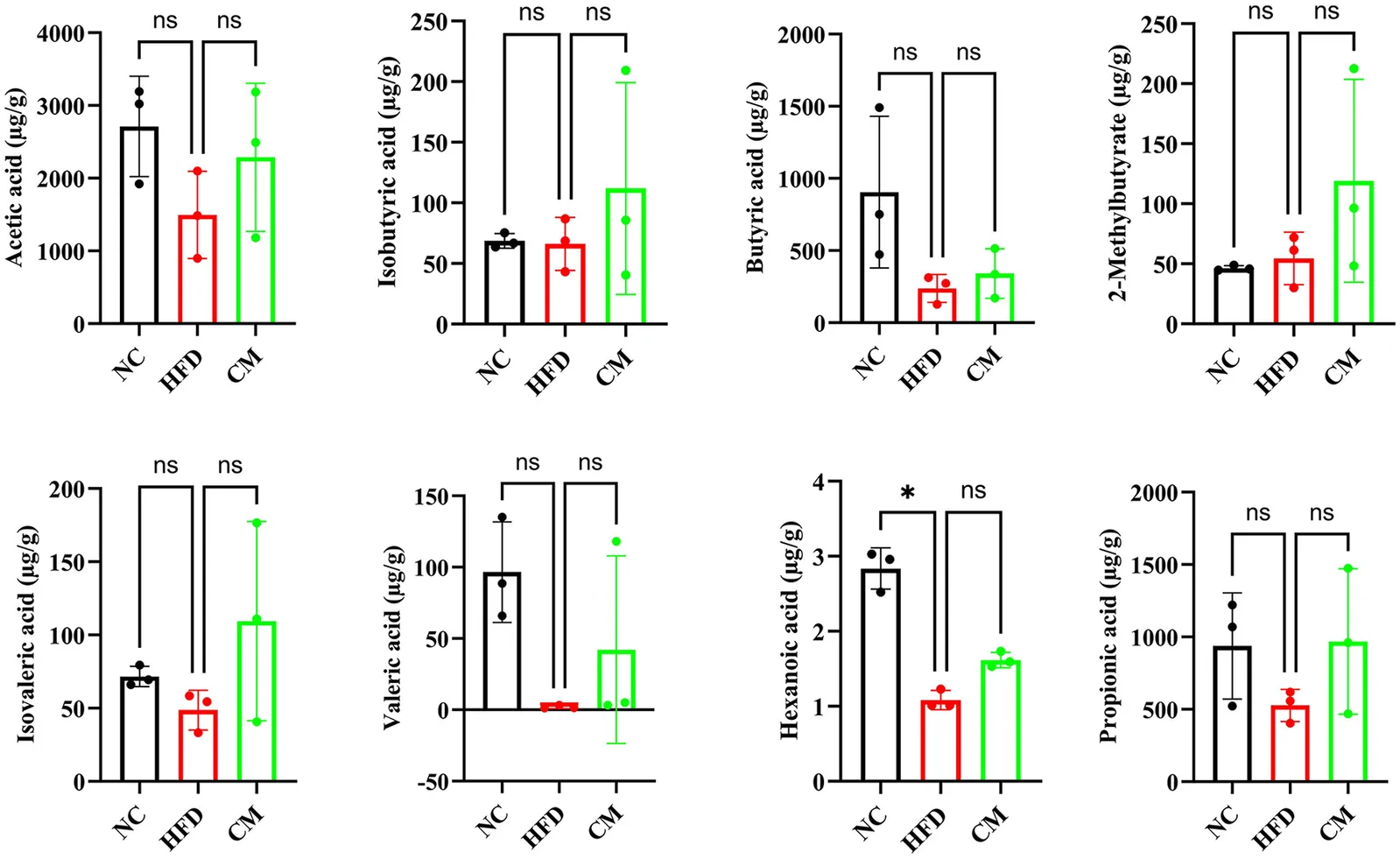
CM altered the colonic SCFA levels in HFD-induced obese mice. Data are expressed as mean ± standard deviation (*n* = 3). Significance was determined by Kruskal–Wallis test with Dunn's multiple comparison test. *p < 0.05. ns, not significant.

### 3.5 CM remodels the hepatic metabolome in HFD-induced obese mice

Principal component analysis of liver metabolomes in positive and negative ion modes demonstrated distinct clustering corresponding to dietary and treatment groups. The HFD group was clearly separated from the NC group. The CM group occupied an intermediate position between NC and HFD, indicating that CM supplementation partially attenuates HFD-induced metabolic shifts in the liver (Supplementary Figure S3A and Supplementary Figure S3B).

Pie chart analysis of the liver metabolome shows the distribution of identified metabolites by chemical class. In both positive-ion and negative-ion modes, lipids and lipid-like molecules constituted the largest proportion of metabolites. Organic acids and their derivatives were the second-most abundant class. Other major categories included nucleosides, nucleotides, analogs, amino acids, peptides, and carbohydrates/carbohydrate conjugates (Figures S3C and D). The classification profiles were notably consistent across ionization modes, highlighting a similar metabolic landscape under both analytical conditions.

Analysis of differential metabolites in the liver metabolome was conducted across three groups. In the positive-ion mode, comparison of HFD vs. NC revealed 443 differential metabolites, indicating substantial metabolic alterations induced by the HFD. CM intervention resulted in 170 differential metabolites between CM and HFD groups, suggesting modulation of the HFD-disturbed metabolome. Furthermore, 479 differential metabolites were identified between the CM and NC groups (Supplementary Figure S3E). In the negative-ion mode, HFD vs. NC comparison showed 244 differential metabolites. In comparison, CM vs. HFD exhibited 158 differential metabolites, reflecting a pronounced metabolic response to CM treatment. Additionally, 265 differential metabolites were detected between CM and NC (Supplementary Figure S3F). Together, these results demonstrate that HFD feeding significantly perturbs the hepatic metabolome, and CM intervention notably modulates these metabolic changes.

KEGG enrichment analysis of hepatic differential metabolites revealed distinct alterations in metabolic pathways between CM-treated and HFD mice in both positive and negative ion modes. In the positive ion mode, pathways enriched included lipid metabolism (e.g., sphingolipid signaling pathway and arachidonic acid metabolism), amino acid metabolism (e.g., phenylalanine/tyrosine/tryptophan and arginine biosynthesis), cellular processes (e.g., apoptosis, necroptosis, platelet activation), and pathways related to oxidative stress and inflammation (e.g., glutathione metabolism, inflammatory mediator regulation of TRP channels) (Figure 5A). This suggests that CM intervention may influence HFD-induced disturbances in lipid homeostasis, inflammation, and cellular stress. Negative-ion mode analysis indicated broad metabolic reprogramming, with a wide range of pathways significantly enriched, reflecting extensive systemic metabolic modulation following CM treatment (Figure 5B). Together, these results highlight the multi-pathway regulatory role of CM in modulating hepatic metabolism, particularly in pathways linked to lipid dysregulation, oxidative stress, and inflammatory responses under HFD conditions.

**Figure 5:**
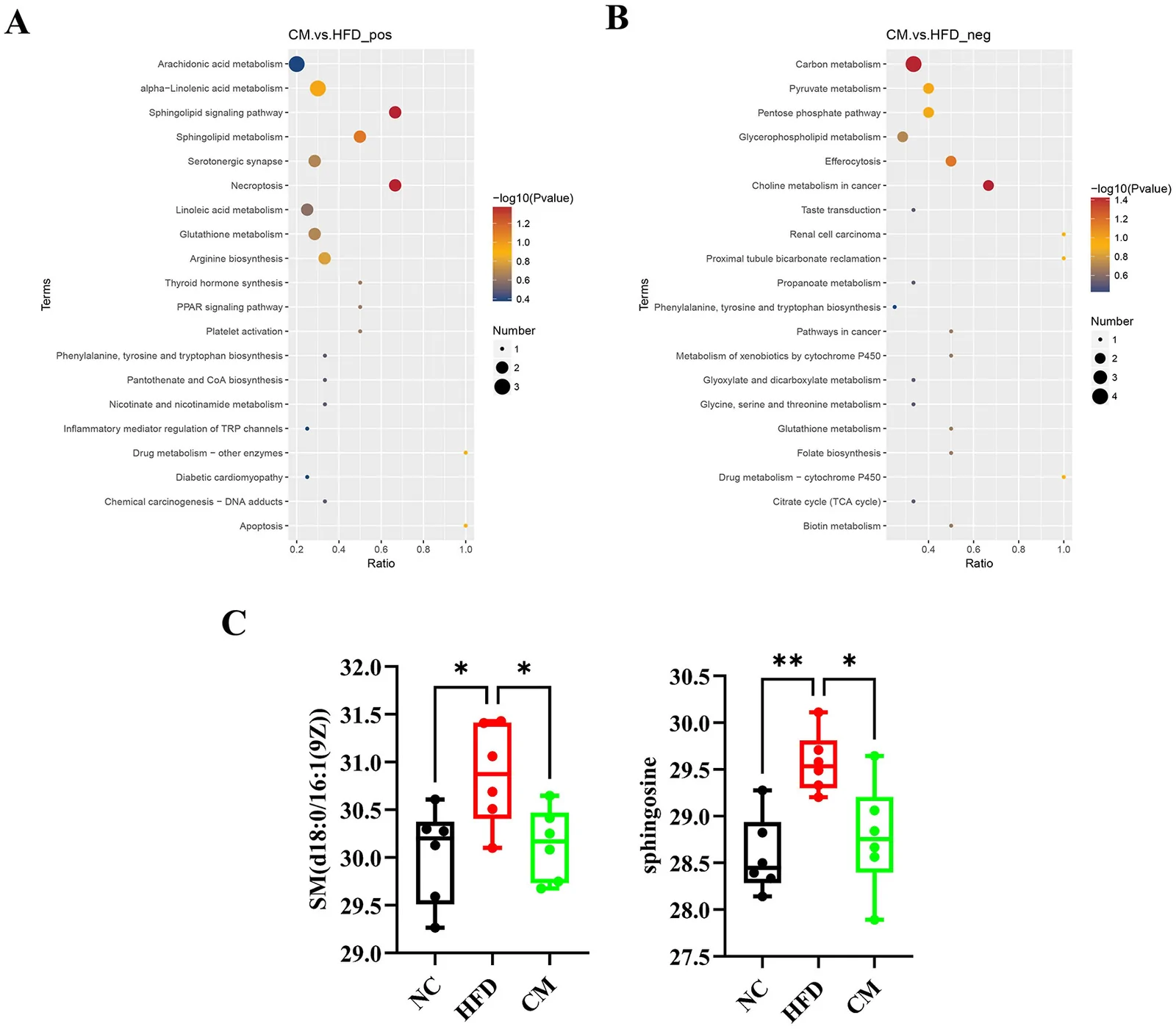
CM altered the hepatic metabolome in HFD-induced obese mice. **(A)** KEGG pathway enrichment scatter plot in positive ion mode. **(B)** KEGG pathway enrichment scatter plot in negative ion mode. **(C)** Abundance of SM(d18:0/16:1(9Z)) and sphingosine. Data are expressed as mean ± standard deviation (*n* = 6). Significance was determined by one-way ANOVA with Tukey's *post hoc* test. *p < 0.05, **p < 0.01. ns, not significant.

Two differential metabolites, SM(d18:0/16:1(9Z)) and sphingosine, were identified in liver tissue among the NC, HFD, and CM groups. Compared with the NC group, both metabolites were elevated in the HFD group. Notably, CM treatment significantly reversed these increases, reducing the abundance of SM(d18:0/16:1(9Z)) and sphingosine in HFD-fed mice (Figure 5C). These findings suggest that CM intervention may ameliorate HFD-induced disturbances in sphingolipid metabolism.

### 3.6 CM remodels the hepatic transcriptome in HFD-induced obese mice

PCA of the liver transcriptome clearly separated the experimental groups. PC1 (20.06%) and PC2 (19.44%) revealed distinct clustering of NC samples, while HFD samples formed a separate cluster. CM samples were positioned between the NC and HFD groups, shifted toward the NC cluster, indicating a partial reversal of HFD-induced transcriptomic changes by CM treatment (Supplementary Figure S4A).

Volcano plots were generated to visualize differentially expressed genes (DEGs) in the liver transcriptome for each comparison, using thresholds of |log_2_FoldChange| ≥ 1 and *p*-value ≤ 0.05. HFD feeding induced extensive transcriptional changes, with many genes significantly up- or downregulated compared to the NC group, reflecting broad metabolic dysregulation (Figure 6A). CM intervention significantly modulated 613 genes compared to the HFD group. Among these, 181 genes were upregulated, and 432 were downregulated. At the same time, the majority of transcripts (25,825) remained unchanged (Figure 6B). This targeted transcriptional response suggests that CM treatment partially reverses certain HFD-induced alterations in gene expression. A total of 271 differentially expressed genes (DEGs) were common to both the HFD vs. NC and CM vs. HFD comparisons, suggesting that CM intervention modulated this subset of genes that were initially altered by the HFD (Supplementary Figure S4B).

**Figure 6:**
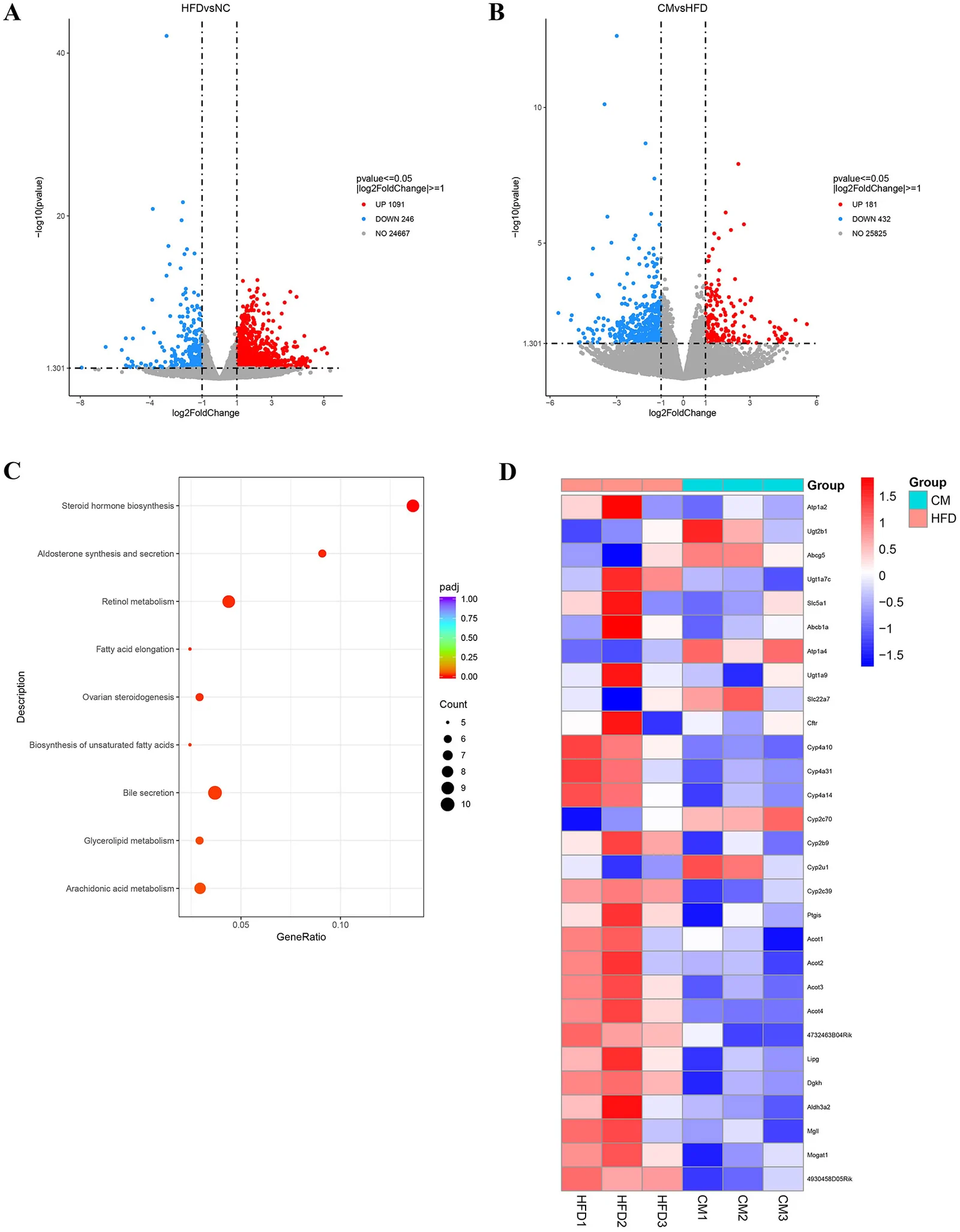
CM altered the hepatic transcriptome in HFD-induced obese mice. **(A)** Volcano plot of HFD vs. NC. **(B)** Volcano plot of CM vs. HFD. **(C)** KEGG pathway enrichment analysis of DEGs. **(D)** Heat map of DEGs related to lipid metabolism.

To investigate the lipid-modulating effects of CM in HFD-fed mice, KEGG enrichment analysis was performed on liver transcriptome data. Several lipid metabolism-related pathways were significantly enriched in the CM vs. HFD comparison, including biosynthesis of unsaturated fatty acids, bile secretion, glycerolipid metabolism, and arachidonic acid metabolism (Figure 6C). These pathways are closely involved in lipid homeostasis and metabolic regulation.

To visualize the expression patterns of DEGs in the liver transcriptome of HFD-fed mice treated with CM, clustering analysis was performed. The heatmap illustrates distinct expression profiles between the CM and HFD groups for genes involved in key metabolic pathways. For genes associated with bile secretion, *Ugt2b1, Abcg5, Atp1a4*, and *Slc22a7* were upregulated, while *Atp1a2, Ugt1a7c, Slc5a1, Abcb1a, Ugt1a9*, and *Cftr* were downregulated in the CM group compared to the HFD group. In the arachidonic acid metabolism pathway, *Cyp2c70* and *Cyp2u1* showed increased expression, whereas *Cyp4a10, Cyp4a31, Cyp4a14, Cyp2b9, Cyp2c39*, and *Ptgis* were decreased following CM treatment. Regarding fatty acid elongation and biosynthesis of unsaturated fatty acids, all identified genes—*Acot3, Acot4, 4732463B04Rik, Acot2*, and *Acot1*—were downregulated in the CM group. Similarly, genes involved in glycerolipid metabolism, including *Lipg, Dgkh, Aldh3a2, Mgll, 4930458D05Rik*, and *Mogat1*, were also downregulated in CM-treated mice (Figure 6D). These clustering results indicate that CM intervention modulates the expression of genes involved in bile secretion, arachidonic acid metabolism, fatty acid metabolism, and glycerolipid metabolism, suggesting a potential role in alleviating HFD-induced hepatic lipid dysregulation.

### 3.7 Correlation analysis between gut microbial genera and obesity-related parameters following CM intervention

To explore the potential relationship between gut microbiota composition and obesity-related phenotypes, Spearman correlation analysis was performed between the relative abundance of six microbial genera and metabolic parameters in HFD-fed mice treated with CM. The results revealed that the abundance of *unclassified [Eubacterium]_coprostanoligenes_group* was significantly negatively correlated with eWAT weight (*r* = −0.846, *p* < 0.01), iWAT weight (*r* = −0.818, *p* < 0.01), TG levels (*r* = −0.673, *p* < 0.05), and the AUC of OGTT (*r* = −0.580, *p* < 0.05), suggesting a potential protective role against adiposity and dyslipidemia. In contrast, *Faecalitalea* abundance showed significant positive correlations with iWAT weight (*r* = 0.685, *p* < 0.05) and TG levels (*r* = 0.637, *p* < 0.05), indicating a possible association with increased fat accumulation and hypertriglyceridemia. No significant correlations were observed between the measured parameters and *Mucispirillum, Escherichia-Shigella, Bacteroides*, or *Parabacteroides*. However, *Parabacteroides* tended to be negatively correlated with BW and adipose tissue weights, though not reaching statistical significance (Figure 7 and Supplementary Figure S5). These findings suggest that CM intervention may modulate certain gut microbiota taxa, such as *unclassified [Eubacterium]_coprostanoligenes_group* and *Faecalitalea*, which are closely linked to improvements in obesity-related metabolic traits.

**Figure 7:**
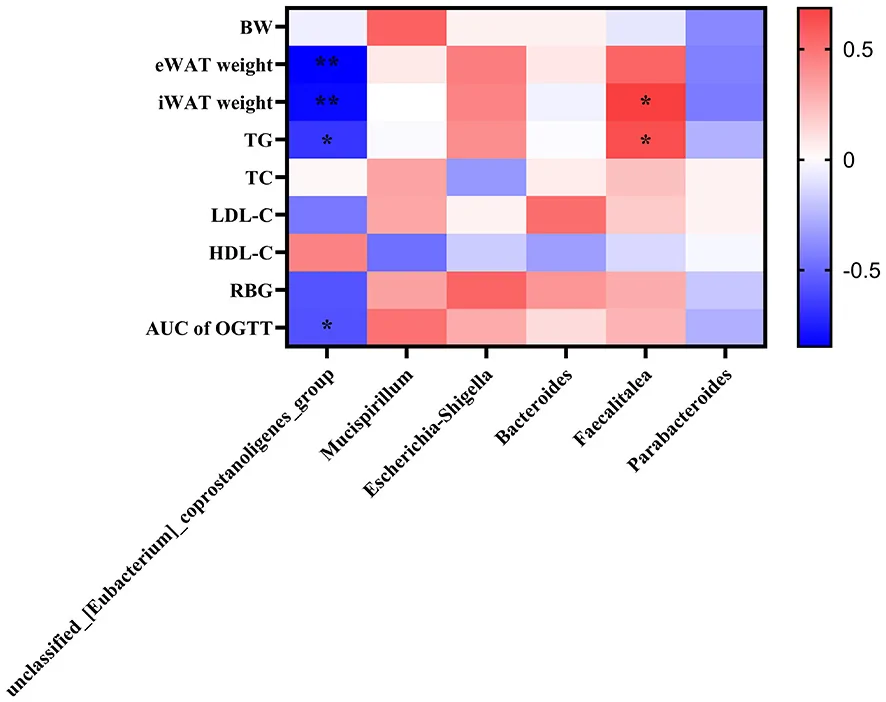
Correlation analysis between gut microbiota and obesity-related indicators. *p < 0.05 and ** *p* < 0.01.

### 3.8 Correlation analysis between gut microbial genera and SCFAs following CM intervention

To investigate the potential impact of CM intervention on microbial metabolic function, Spearman correlation analysis was performed between the relative abundances of six gut microbial genera and SCFA levels in HFD-fed mice. The analysis revealed several significant correlations between certain bacterial genera and SCFA concentrations. *Mucispirillum* abundance was significantly negatively correlated with butyric acid levels (*r* = −0.845, *p* < 0.05), suggesting a potential inhibitory relationship with this key SCFA. *Bacteroides* showed a strong negative correlation with valeric acid (*r* = −0.829, *p* < 0.05). Notably, *unclassified [Eubacterium]_coprostanoligenes_group* exhibited a strong positive correlation with hexanoic acid (*r* = 0.714), although this did not reach statistical significance, it may indicate a trend toward enhanced production of this medium-chain fatty acid. In contrast, *Faecalitalea* tended to be negatively correlated with multiple SCFAs, including acetic, butyric, and hexanoic acids, mirroring the opposite pattern of *unclassified [Eubacterium]_coprostanoligenes_group*. No significant correlations were observed for *Escherichia-Shigella* or *Parabacteroides* with any of the measured SCFAs. However, *Parabacteroides* showed a moderate positive correlation with butyric acid (*r* = 0.486) and a negative trend with valeric and hexanoic acids (Figure 8). These findings suggest that CM intervention may modulate gut microbial composition, particularly influencing taxa such as *Mucispirillum, Bacteroides*, and *unclassified [Eubacterium]_coprostanoligenes_group*, which are closely associated with alterations in SCFA profiles, potentially contributing to improved metabolic health.

**Figure 8:**
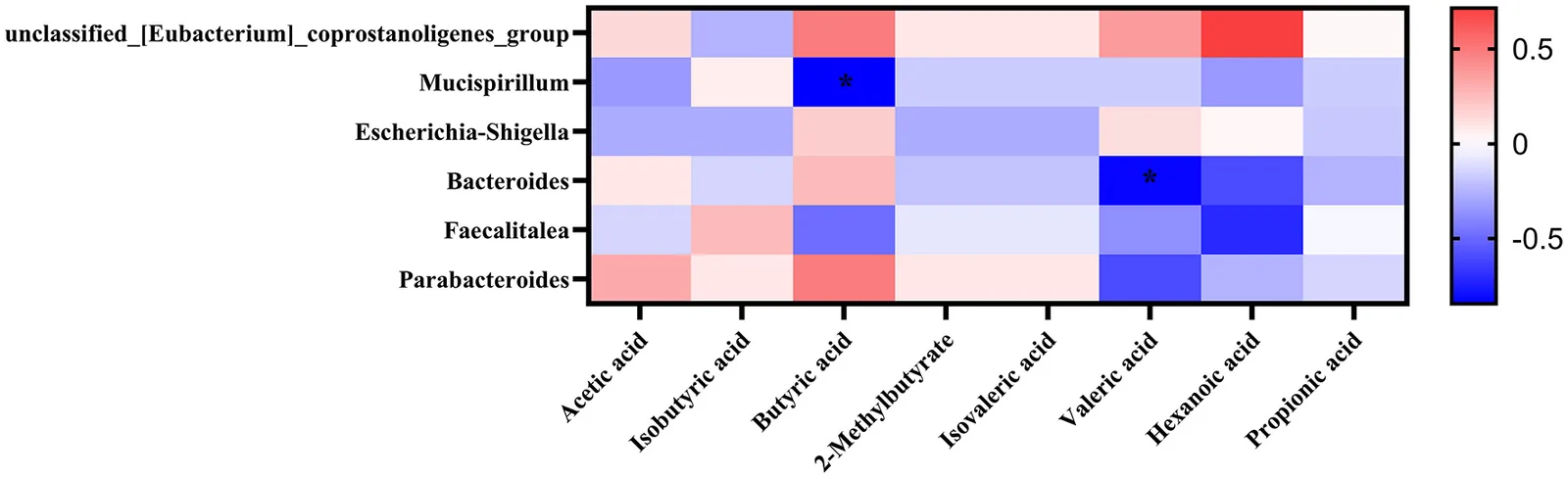
Correlation analysis between gut microbiota and colonic SCFAs. *p < 0.05 and ** *p* < 0.01.

### 3.9 Correlation analysis between gut microbial genera and hepatic metabolites following CM intervention

To investigate the relationship between gut microbiota and hepatic metabolism following CM intervention, Spearman correlation analysis was performed between six microbial genera and liver metabolite levels in HFD-fed mice. The results showed that *unclassified [Eubacterium]_coprostanoligenes_group* was significantly positively correlated with multiple hepatic metabolites involved in amino acid metabolism (e.g., L-ornithine), glutathione metabolism (e.g., glutathione disulfide, S-hydroxymethylglutathione, 5-oxoproline), carbohydrate metabolism (e.g., D-erythrose 4-phosphate, L-malate, sedoheptulose 7-phosphate), and lipid-related metabolites [e.g., PC(20:4/15:0), LPC 18:1]. Conversely, it was negatively correlated with sphingolipids [SM(d18:0/16:1(9Z))], prostaglandin G2, and several unsaturated fatty acids. In contrast, *Escherichia-Shigella* exhibited an opposing pattern, with significant positive correlations with sphingosine, SM(d18:0/16:1(9Z)), and SN-38, and negative correlations with most metabolites positively associated with *unclassified [Eubacterium]_coprostanoligenes_group*, including L-ornithine, glutathione-related metabolites, and various phosphate sugars. *Faecalitalea* showed negative correlations with L-ornithine and several glutathione-related metabolites. In contrast, *Bacteroides, Mucispirillum*, and *Parabacteroides* showed few or no significant correlations with the detected liver metabolites (Figure 9). These findings suggest that CM intervention may modulate certain gut microbiota, particularly *unclassified [Eubacterium]_coprostanoligenes_group* and *Escherichia-Shigella*, which are closely associated with alterations in hepatic sphingolipid, amino acid, glutathione, and carbohydrate metabolism, highlighting a potential microbiota–liver axis in the metabolic benefits of CM.

**Figure 9:**
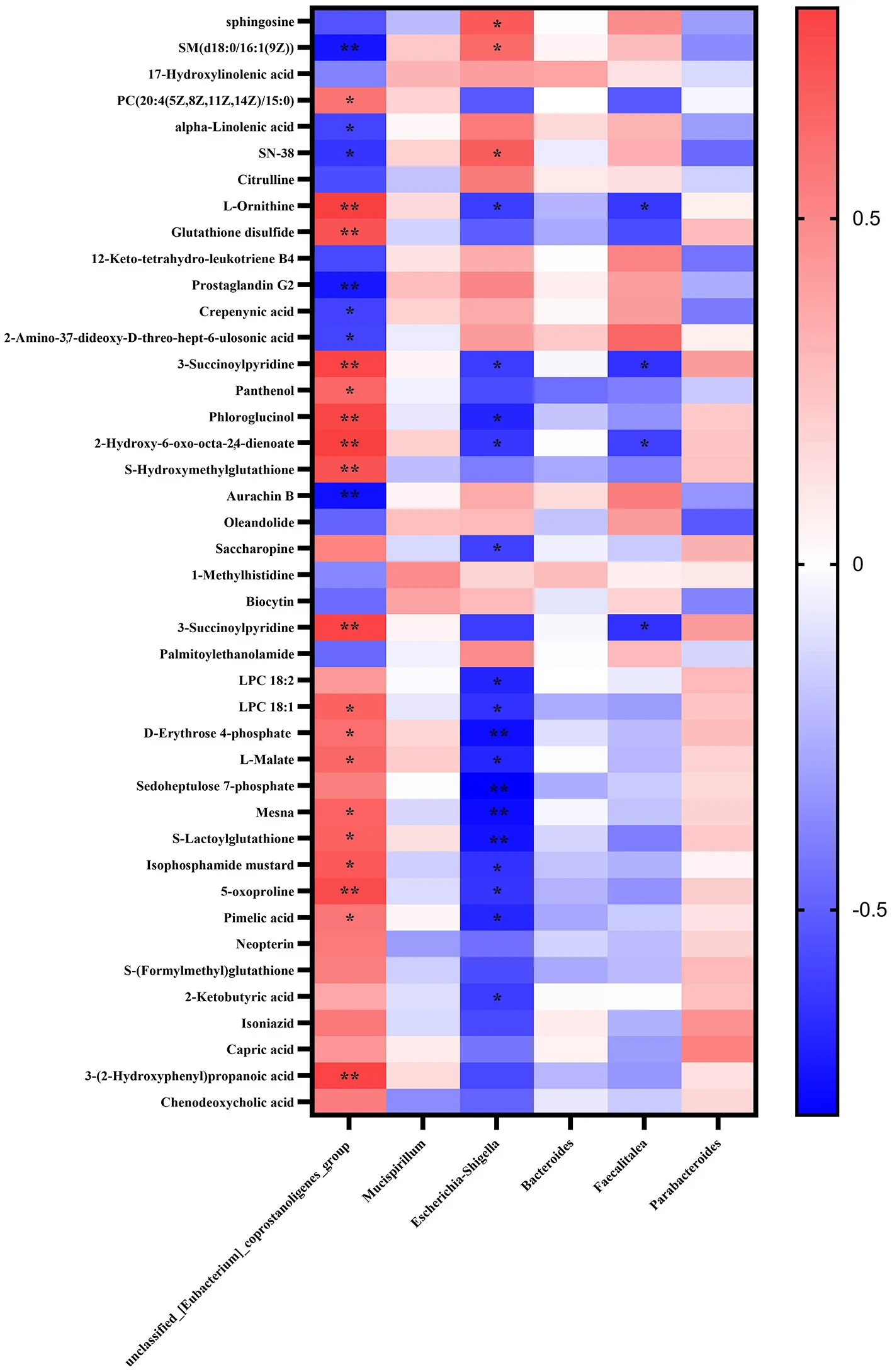
Correlation analysis between gut microbiota and hepatic metabolites. *p < 0.05 and ** *p* < 0.01.

### 3.10 Correlation analysis between gut microbial genera and DEGs related to lipid metabolism following CM intervention

To investigate the potential link between gut microbiota and hepatic gene expression following CM intervention, Spearman correlation analysis was performed between six microbial genera and the expression levels of DEGs involved in lipid metabolism in the liver of HFD-fed mice. The analysis revealed that *unclassified [Eubacterium]_coprostanoligenes_group* was significantly positively correlated with *Cyp2c70* (arachidonic acid metabolism) and negatively correlated with multiple genes across several pathways, including *Cyp4a10, Cyp4a31, Cyp4a14* (arachidonic acid metabolism), *Acot1, Acot2, Acot4, 4732463B04Rik* (fatty acid elongation and unsaturated fatty acid biosynthesis), *Aldh3a2* (glycerolipid metabolism), and *Ugt1a7c* (bile secretion). *Faecalitalea* exhibited an opposing pattern, with significant positive correlations with most genes that were negatively correlated with *unclassified [Eubacterium]_coprostanoligenes_group* (e.g., *Cyp4a10, Cyp4a31, Cyp4a14, Acot1, Acot2, Acot4, Aldh3a2, Ugt1a7c*), and a perfect negative correlation with *Cyp2c70*. *Escherichia-Shigella* showed significant positive correlations with *Slc5a1* (bile secretion) and negative correlations with *Ugt2b1* and *Abcg5* (also bile secretion). *Mucispirillum, Bacteroides*, and *Parabacteroides* showed no statistically significant correlations with the hepatic genes analyzed (Figure 10). These findings suggest that CM intervention may modulate certain gut microbiota—particularly *unclassified [Eubacterium]_coprostanoligenes_group, Faecalitalea*, and *Escherichia-Shigella*—which are closely associated with the expression of hepatic genes involved in bile secretion, arachidonic acid metabolism, fatty acid metabolism, and glycerolipid metabolism.

**Figure 10:**
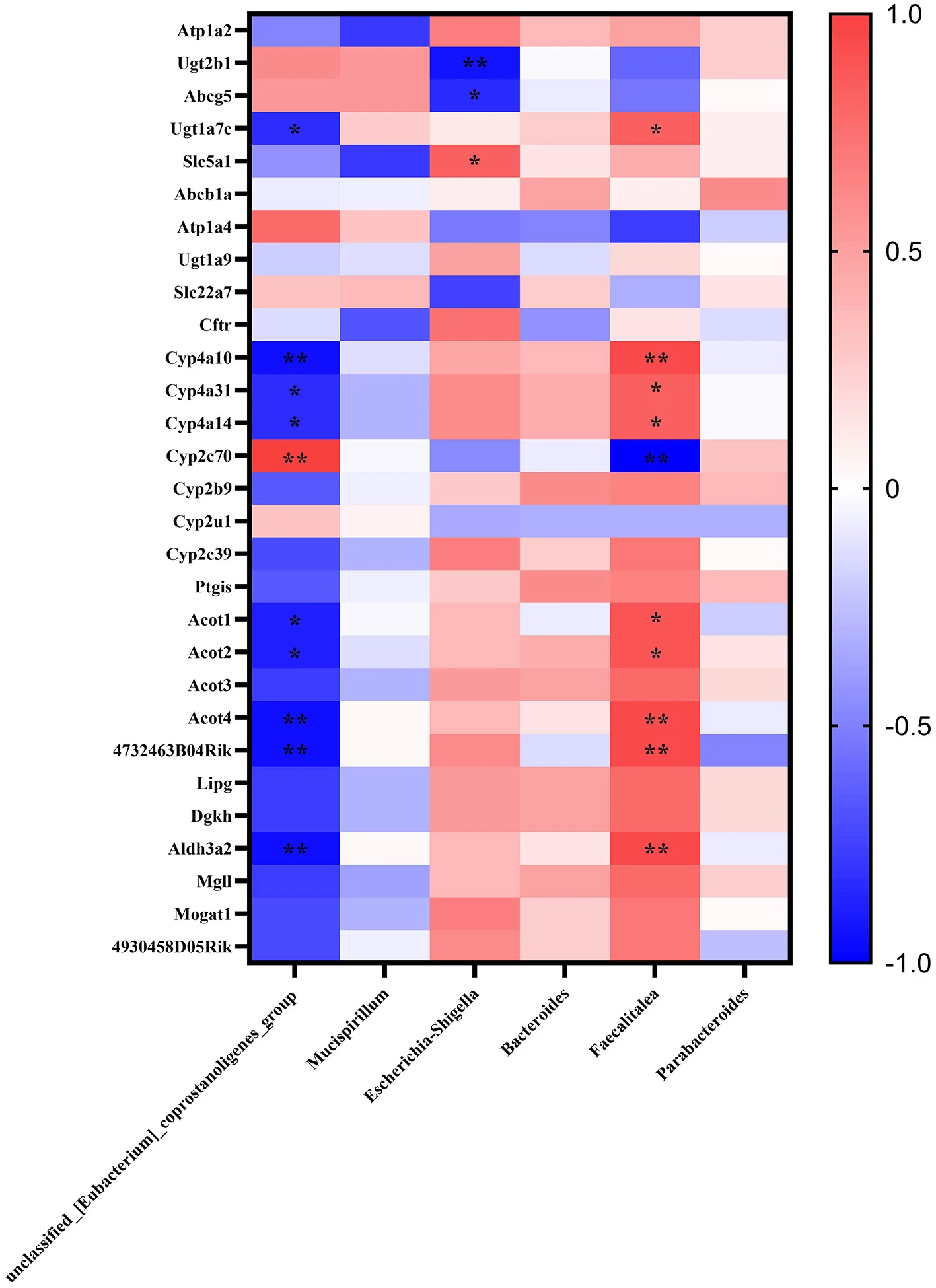
Correlation analysis between gut microbiota and DEGs related to lipid metabolism. *p < 0.05 and ** *p* < 0.01.

## 4 Discussion

This study was designed to explore the protective effects of CM against HFD-induced metabolic syndrome and to examine potential mechanisms involving the gut-liver axis systematically. We hypothesized that the metabolic benefits of CM are mediated through modulation of the gut microbiota and associated metabolites. In support of this hypothesis, our integrated analyses reveal that CM supplementation effectively alleviated major HFD-induced metabolic abnormalities in mice, including BW, impaired glucose tolerance, and dyslipidemia. Importantly, these improvements were accompanied by a moderate modulation of the gut microbial community, with a notable increase in the relative abundance of key bacterial taxa known for producing SCFAs. Correspondingly, intestinal SCFA levels were elevated following CM intervention. Hepatic metabolomic profiling further demonstrated that CM treatment normalized metabolic disturbances induced by HFD, actively restoring pathways involved in lipid, amino acid, and central carbon metabolism. Taken together, these findings provide compelling multi-omics evidence that CM mitigates diet-induced metabolic dysregulation through a coordinated mechanism that reshapes the gut microbiota, enhances microbial SCFA production, and thereby improves hepatic metabolic homeostasis.

Our objective was to investigate the full bioactive potential of CM as it is traditionally consumed in the region. Many of the health-promoting components of CM, such as lactoferrin, lysozyme, immunoglobulins, and insulin-like proteins, are heat-sensitive and are significantly reduced or denatured by conventional pasteurization. Therefore, raw milk was chosen to preserve these native bioactive constituents. Although the use of raw milk maximizes the retention of bioactive factors, we cannot completely exclude the possibility that heat-sensitive enzymes or trace microbial metabolites present in the raw milk contribute to the observed phenotypes.

This study demonstrated that gavage administration of whole CM alleviated HFD-induced metabolic syndrome in mice, as shown by reduced BW, improved oral glucose tolerance, favorable serum lipid profiles, and attenuated hepatic steatosis. These findings directly align with key metabolic outcomes reported for certain CM peptides. The observed improvement in glucose tolerance corroborates the core finding of reduced hyperglycemia in diabetic and HFD-fed mice treated with CM peptides (10, 11). Similarly, the amelioration of dyslipidemia and hepatic fat accumulation is consistent with the improved glucose-lipid metabolism documented for the CM-derived peptide Leucine-Leucine-Proline-Lysine (LLPK) (12). Thus, our results validate that the beneficial metabolic effects of bioactive components, as indicated in focused peptide studies, are effectively delivered and preserved within the complex matrix of whole CM, supporting its role as a functional nutritional intervention.

We explicitly acknowledge that CM induced only a modest overall shift in gut microbiota composition, as evidenced by the substantial overlap between the HFD and CM groups along PC1 (68.43% variance), which primarily captured the dominant effect of the high-fat diet, and the separation that was restricted to PC2 (7.67% variance). We clarify that although certain bacterial taxa were altered, the global restructuring of the community structure was moderate rather than profound. The decrease of Bacteroidota and increase of Pseudomonadota in HFD mice are hallmarks of dysbiosis (13, 14). The restoration of these two phyla by CM is a strong indicator of improved gut ecosystem stability. Evidence indicates that the abundance of *Mucispirillum* is increased in the gut of HFD-fed mice and is closely linked to intestinal inflammation (15–17). The observed reduction in this genus following CM treatment in the present study may therefore suggest a protective effect of CM on the intestinal mucosal barrier in HFD-fed mice. CM administration reduced the abundance of *Escherichia-Shigella* in HFD-fed mice. HFD enriches this pro-inflammatory genus, which disrupts the mucus layer (18). As *Escherichia-Shigella* can penetrate mucus and damage tissue, its reduction suggests that CM preserves intestinal barrier integrity. The increase in *Bacteroides* abundance following CM treatment aligned with previous findings that probiotic interventions (e.g., *L. plantarum*) elevated this genus, thereby enhancing SCFA production. As key SCFA producers, *Bacteroides* ferment dietary polysaccharides, and their enrichment suggested improved metabolic potential of the gut microbiota. The observed decrease in *Faecalitalea* following CM intervention aligns with a meta-analysis by Gong et al., who reported that this genus was significantly enriched in individuals with simple obesity compared to normal-weight controls (19). CM treatment increased the abundance of *Parabacteroides* in HFD-fed mice. This genus, particularly *P. goldsteinii* and *P. johnsonii*, has demonstrated therapeutic potential against obesity through mechanisms including immune modulation, enhanced intestinal barrier function, and promotion of branched-chain amino acid catabolism (20, 21). The elevation of *Parabacteroides* may therefore contribute to CM's anti-obesity effects.

This study investigated the impact of CM on gut health by measuring SCFAs in HFD-fed mice. Our results show that an HFD reduced key SCFAs (acetic, propionic, and butyric acids) compared with the NC group. This reduction likely resulted from a lack of fermentable dietary fiber in the HFD, which deprives gut bacteria of the substrate needed for SCFA production. This depletion is metabolically harmful because these SCFAs are crucial for gut barrier integrity (butyrate), appetite regulation, and energy metabolism (22). Conversely, CM treatment effectively reversed this deficiency, elevating most SCFAs compared to the HFD group. Notably, levels of acetic and propionic acid in the CM group were restored to those seen in the healthy NC group. These findings indicate that CM consumption favorably modulates the gut microbial environment, promoting the recovery of SCFA-producing bacterial communities and their metabolic output. While this modulatory effect may contribute to the metabolic benefits of CM, the current data do not allow us to definitively classify CM as a prebiotic, as the precise substrates and mechanisms underlying this microbial shift remain to be elucidated through targeted studies. A key finding of our study was the distinct pattern of hexanoic acid across groups: highest in the NC group, lowest in the HFD group, and partially restored in the CM-treated group. This suggests hexanoic acid may be a sensitive marker of gut health and dietary intervention. The reduction of hexanoic acid in HFD-fed mice is significant because recent research has identified hexanoic acid as a free fatty acid with notable anti-obesity properties. It suppresses HFD-induced obesity and fat accumulation, in part by inhibiting lipogenesis. Therefore, the low levels observed in our HFD group may contribute to the obese phenotype. The partial restoration of hexanoic acid by CM treatment is particularly meaningful. This is because hexanoic acid is more potent than butyric acid in maintaining glucose homeostasis and improving insulin sensitivity and hyperinsulinemia under HFD conditions (23). The SCFA results should be interpreted with caution due to the small sample size and the associated potential for high variability. Despite this limitation, the observed trends in SCFA levels (particularly the changes in acetate, propionate, and butyrate) are largely consistent with the alterations in gut microbiota composition and metabolic phenotypes, suggesting biological relevance.

The present study demonstrated that CM intervention significantly reversed HFD-induced elevations in hepatic sphingosine and SM(d18:0/16:1(9Z)), two key metabolites enriched in the sphingolipid signaling pathway, sphingolipid metabolism, and necroptosis. Our finding that HFD feeding increases hepatic sphingolipid levels aligns with the established literature, which shows that sphingolipid accumulation is a hallmark of obesity-related metabolic dysfunction. Green et al. reported that ceramides and bioactive sphingolipids accumulate in obese tissues, contributing to insulin resistance and hepatic steatosis (24). The elevated sphingosine observed in HFD mice serves as a precursor for both ceramide and sphingosine-1-phosphate (S1P), suggesting enhanced flux through sphingolipid metabolic pathways. The reversal of these elevations by CM treatment is particularly significant. Juchnicka et al. emphasized that the balance between ceramide, sphingosine, and S1P is critical for metabolic homeostasis, with disruptions contributing to inflammation and insulin resistance (25). By reducing hepatic sphingosine levels, CM may help restore this equilibrium and mitigate lipotoxic stress. Kajita et al. further highlighted that S1P signaling regulates obesity and glucose homeostasis, noting that ceramide accumulation impairs insulin sensitivity in the liver and muscle (26). The identification of sphingolipid signaling as a key target of CM intervention supports the concept that nutritional components can modulate sphingolipid-regulated physiology. Voß et al. comprehensively reviewed how dietary factors influence sphingolipid profiles and metabolic disease risk (27). CM's unique bioactive composition may directly modulate hepatic sphingolipid metabolism. Additionally, enrichment of the necroptosis pathway suggests that CM may protect against HFD-induced hepatocyte cell death, as sphingolipids are known regulators of cell death pathways. CM intervention ameliorates HFD-induced disturbances in hepatic sphingolipid metabolism, representing a potential nutritional strategy for obesity-related metabolic dysfunction.

In the present study, transcriptomic analysis revealed that CM intervention significantly modulated hepatic gene expression profiles in HFD-fed mice. The downregulation of all identified genes in the fatty acid elongation and unsaturated fatty acid biosynthesis pathways, specifically the acyl-CoA thioesterase family members *Acot1, Acot2, Acot3, Acot4*, and *4732463B04Rik*, suggests that CM may influence hepatic fatty acid metabolism. ACOTs catalyze the hydrolysis of acyl-CoAs to free fatty acids and CoA, thereby regulating intracellular fatty acid levels (28). However, as transcriptional changes do not directly reflect enzymatic activity or metabolic flux, the downregulation of these genes should be interpreted as indicative of transcriptional reprogramming of lipid metabolism rather than definitive evidence of reduced fatty acid synthesis. Further studies measuring enzymatic activity or fatty acid flux are needed to confirm this hypothesis. A particularly striking finding was the differential regulation of cytochrome P450 (CYP) family genes within the arachidonic acid metabolism pathway. CM treatment downregulated *Cyp4a10, Cyp4a31*, and *Cyp4a14*. The suppression of multiple Cyp4a family members is of considerable interest in the context of metabolic dysfunction-associated steatotic liver disease (MASLD), as hepatic CYP4A expression is induced in multiple murine models of NAFLD and in patients with the disease (29, 30). However, since 20-hydroxyeicosatetraenoic acid (20-HETE) levels were not directly measured in this study, these transcriptomic changes alone cannot establish altered 20-HETE signaling. Therefore, the downregulation of these genes should be regarded as a potential indication that CM may modulate the CYP4A/20-HETE axis, a hypothesis that warrants further investigation through targeted lipidomic analyses. The downregulation of genes in the glycerolipid metabolism pathway, including *Mogat1* and *Mgll*, further suggests that CM may influence hepatic lipid synthesis and storage at the transcriptional level, although direct measurements of glycerolipid species or enzymatic activities would be required to confirm functional consequences.

Regarding bile secretion, the upregulation of *Abcg5* is noteworthy, as it encodes a canalicular cholesterol transporter that forms an obligate heterodimer with *Abcg8* to mediate cholesterol efflux from hepatocytes into bile (31). While increased *Abcg5* transcript levels suggest a potential enhancement of hepatobiliary cholesterol excretion capacity, direct measurements of biliary cholesterol secretion or reverse cholesterol transport were not performed in this study. Therefore, this finding should be interpreted as transcriptional regulation favoring cholesterol elimination rather than conclusive evidence of enhanced reverse cholesterol transport. In summary, the hepatic transcriptomic analysis indicates that CM exerts multimodal transcriptional effects on lipid metabolism-related pathways in HFD-fed mice, including the modulation of genes involved in fatty acid metabolism, arachidonic acid metabolism, glycerolipid metabolism, and cholesterol transport. It should be emphasized that these mechanistic inferences are based on gene expression changes and have not been functionally validated at the protein, enzymatic, or metabolite levels in this study. Future studies incorporating targeted metabolomics, enzymatic activity assays, and metabolic flux analyses are warranted to confirm the functional significance of these transcriptional changes.

The present study found that *unclassified [Eubacterium]_coprostanoligenes_group* abundance was significantly negatively correlated with eWAT and iWAT weights, TG levels, and OGTT AUC in HFD-fed mice treated with CM, suggesting a protective role against obesity. These findings are consistent with previous studies. Zhang et al. reported that *[Eubacterium]_coprostanoligenes_group* was negatively correlated with body weight, serum TC, TG, LDL-C, and hepatic lipids in hyperlipidemic rats (32). Liu et al. also showed that *Eubacterium_coprostanoligenes_group_unclassified* negatively correlated with body weight, LDL-C, TC, and adipocyte enlargement in HFD-fed mice (33).

This study investigated the relationship between gut microbiota and hepatic metabolism following CM intervention in HFD-fed mice. Spearman correlation analysis revealed distinct and opposing association patterns between certain microbial genera and hepatic metabolites, providing insights into potential mechanisms underlying CM's hepatoprotective effects via the gut–liver axis (34). The *unclassified [Eubacterium]_coprostanoligenes_group* showed significant positive correlations with multiple hepatic metabolites involved in amino acid metabolism (L-ornithine), glutathione metabolism (glutathione disulfide, S-hydroxymethylglutathione, 5-oxoproline), and carbohydrate metabolism (D-erythrose 4-phosphate, L-malate, sedoheptulose 7-phosphate). Members of the *Eubacterium coprostanoligenes group* are recognized for their cholesterol-reducing capabilities (32), but our findings suggest broader metabolic influence. The positive association with glutathione-related metabolites is particularly noteworthy, as glutathione is critical for hepatic antioxidant defense. This aligns with studies demonstrating that CM enhances liver antioxidant capacity and modulates gut microbiota in mice (8, 35). Conversely, this genus was negatively correlated with sphingolipids [SM(d18:0/16:1(9Z))], prostaglandin G2, and unsaturated fatty acids, suggesting potential protection against lipotoxicity and inflammatory signaling (36, 37). In stark contrast, *Escherichia-Shigella* showed an opposing correlation pattern, with significant positive associations with sphingosine, sphingomyelin, and SN-38, and negative correlations with most metabolites that were positively linked to the *Eubacterium group*. *Escherichia-Shigella* species are Gram-negative bacteria whose expansion is a hallmark of gut dysbiosis in metabolic diseases (38). These bacteria produce lipopolysaccharide (LPS), which can trigger hepatic inflammation via TLR4 signaling, promoting insulin resistance and steatosis (39). Previous studies have shown that CM consumption can reduce potentially harmful bacteria, including Escherichia species, in diabetic and liver disease models (40, 41). This study demonstrates that CM intervention in HFD-fed mice is associated with distinct correlation patterns between certain gut microbial genera and hepatic metabolites. The *unclassified Eubacterium_coprostanoligenes_group* emerges as a candidate beneficial mediator linked to antioxidant defense and energy metabolism, while *Escherichia-Shigella* shows an opposing pattern consistent with a pro-inflammatory role.

We employed Spearman's correlation analysis to examine the relationship between CM-induced changes in gut microbiota and hepatic gene expression in HFD-fed mice. Distinct correlation patterns were observed between certain bacterial genera and genes involved in lipid metabolism, bile secretion, and arachidonic acid metabolism, highlighting a potential gut–liver axis in CM's metabolic benefits. The *unclassified [Eubacterium]_coprostanoligenes_group* showed a positive correlation with *Cyp2c70* (arachidonic acid metabolism) and negative correlations with genes in fatty acid elongation, unsaturated fatty acid biosynthesis, glycerolipid metabolism, and bile secretion, including *Cyp4a10, Cyp4a31, Cyp4a14, Acot1/2/4, Aldh3a2*, and *Ugt1a7c*. CYP4A enzymes are often upregulated in metabolic disorders (42), and ACOTs are key regulators of hepatic lipid homeostasis (43, 44). This suggests that this bacterium may help ameliorate steatosis by suppressing lipogenic pathways. *Faecalitalea* exhibited an opposing pattern. It positively correlated with most genes negatively linked to the Eubacterium group (e.g., *Cyp4a10, Cyp4a31, Cyp4a14, Acot1/2/4, Aldh3a2, Ugt1a7c*) and negatively with *Cyp2c70*. ACOTs maintain acyl-CoA homeostasis and facilitate fatty acid oxidation (45). This opposing correlation suggests that CM may shift the microbial ecosystem, thereby favorably reprogramming hepatic gene expression. *Escherichia-Shigella* correlated with bile secretion genes. It positively correlated with *Slc5a1* and negatively with *Ugt2b1* and *Abcg5*. Bile components can stimulate bacterial efflux pumps (46), and CM is known to modulate gut microbiota in obese models (47). This suggests a bidirectional interaction influencing bile acid metabolism. Collectively, these findings support that CM modulates certain gut microbiota—*unclassified [Eubacterium]_coprostanoligenes_group, Faecalitalea*, and *Escherichia-Shigella*—which are closely associated with hepatic genes regulating bile secretion, arachidonic acid, fatty acid, and glycerolipid metabolism. Although the multi-omics integration revealed consistent and biologically plausible associations between gut microbiota, SCFAs, and hepatic lipid metabolism, the relatively small sample size (particularly *n* = 3 for SCFAs and transcriptomics) warrants caution in overinterpreting these findings. Larger independent cohorts will be needed to validate the key associations identified here.

While our multi-omics analysis mechanistically links CM supplementation to remodeling of the gut microbiota and hepatic lipid metabolism, the distinctive nutritional and bioactive profile of Bactrian CM offers a plausible biological context for these findings. Compared to bovine and other ruminant milks, CM is uniquely rich in vitamin C, possesses a distinct fatty acid profile with a higher proportion of long-chain unsaturated fatty acids, and contains appreciable levels of insulin-like protein, lactoferrin, and immunoglobulins (48). These components may rationally participate in the metabolic reprogramming we observed. For instance, the unique fatty acid composition could serve as a selective substrate for certain gut microbial taxa, potentially driving the beneficial shifts in community structure identified in our analysis. Concurrently, the elevated vitamin C and insulin-like protein content could synergistically attenuate hepatic oxidative stress and inflammation, providing a molecular foundation for the favorable remodeling of hepatic lipid metabolism. Furthermore, the abundant lactoferrin and immunoglobulins may directly shape the intestinal ecosystem by suppressing pathobionts and fostering a mucosal environment conducive to beneficial bacteria. However, these mechanistic links must be interpreted with caution, as they represent supportive hypothetical frameworks for the observed multi-omics changes rather than conclusive evidence of causality specific to CM.

Several important limitations of this study must be explicitly acknowledged. First, the absence of a pair-fed control group prevents us from fully segregating the effects of CM-specific bioactivities from the secondary effects of reduced energy intake. While our multi-omics analysis strongly suggests that CM induces unique microbial and hepatic signatures distinct from simple caloric restriction, future studies utilizing isocaloric pair-feeding designs are necessary to validate the direct, calorie-independent mechanisms of CM. Second, our study lacks a conventional milk control (e.g., bovine or goat milk), which limits our ability to determine whether the observed effects are specific to CM or reflect more general effects of milk supplementation. Although CM differs substantially from bovine milk in protein and lipid composition, and previous studies have suggested unique bioactivities of CM components, future comparative studies with other milk types are warranted to establish CM-specific mechanisms. Third, it is important to note that the relationships among gut microbiota, SCFAs, hepatic transcriptomic changes, and metabolic improvements observed in this study are correlative. Our data do not establish a causal role for the gut–liver axis in mediating the beneficial effects of CM. Future studies employing fecal microbiota transplantation, microbiota depletion, or other mechanistic interventions are required to determine whether and how gut microbiota causally contribute to the metabolic benefits of CM. Fourth, our experiments were conducted exclusively in male mice, which limits the generalizability of these findings to females. Given that sex-specific differences in metabolic regulation and gut microbial composition are well-documented, future investigations incorporating both sexes are essential. Fifth, our mechanistic insights are derived solely from hepatic transcriptomic data, and protein-level validation of key differentially expressed genes, such as through western blotting or immunohistochemistry, was not performed. Finally, although our findings are encouraging, they were obtained from a diet-induced obesity mouse model. Considering the variability in CM composition arising from geographical region, species, and processing methods, as well as the physiological differences between mice and humans, the direct translatability of these results to human obesity and metabolic syndrome remains uncertain. We therefore call for well-designed clinical studies to validate these findings in human populations.

## 5 Conclusion

This study demonstrates that CM intervention effectively counteracts HFD-induced metabolic syndrome in mice, as evidenced by reduced body weight, improved glucose tolerance, and alleviated dyslipidemia and hepatic steatosis. Using a multi-omics approach, our findings reveal that these metabolic improvements were accompanied by marked alterations in the gut microbiota and hepatic metabolic profiles. Specifically, CM treatment restructured the gut microbiota by enriching SCFA-producing taxa (e.g., *Bacteroides, Parabacteroides*) and reducing pro-inflammatory genera (e.g., *Escherichia-Shigella, Faecalitalea*), which corresponded with restored levels of key SCFAs. Integrated multi-omics analyses further revealed significant associations between these microbial shifts and normalized hepatic metabolomic profiles—particularly in sphingolipid and amino acid metabolism—as well as modulated hepatic transcriptomic pathways involved in lipid synthesis, fatty acid elongation, and bile secretion. Notably, the *unclassified [Eubacterium]_coprostanoligenes_group* emerged as a bacterial taxa showing positive correlations with antioxidant pathways and negative correlations with lipotoxic metabolites, suggesting a potential contributing role. Collectively, while the current experimental design does not permit definitive establishment of causality, these convergent findings are consistent with a model in which CM-induced modulation of the gut microbiota and its metabolic output (e.g., SCFAs) may contribute to the restoration of hepatic metabolic homeostasis. Future studies employing causality-establishing approaches—such as fecal microbiota transplantation, germ-free mouse models, or selective depletion of specific microbial taxa—are warranted to validate the proposed gut–liver axis mechanism.

## Data Availability

The data presented in the study are deposited in the NCBI BioProject repository (https://www.ncbi.nlm.nih.gov/bioproject), accession number PRJNA1538140.

## Supplementary material

Supplementary Figure S1 Experimental timeline.

Supplementary Figure S2 Gut microbiota. **(A)** NMDS of gut microbiota. **(B)** Relative abundance of bacteria at the phylum level. **(C)** Relative abundance of bacteria at the genus level.

Supplementary Figure S3 Hepatic metabolome. **(A)** PCA in positive ion mode. **(B)** PCA in negative ion mode. **(C)** Metabolite classification and abundance in positive ion mode. **(D)** Metabolite classification and abundance in negative ion mode. **(E)** Venn diagram in positive ion mode. **(F)** Venn diagram in negative ion mode.

Supplementary Figure S4 Hepatic transcriptome. **(A)** PCA of transcriptome. **(B)** Venn diagram.

Supplementary Figure S5 Scatter plots showing correlations (Figure 7).

Supplementary Table S1 Camel milk composition.

Supplementary Table S2 Calibration curve for SCFAs.

Supplementary Table S3 Analysis of BW measurements in each group.

Supplementary Table S4 Food intake in each group.

Supplementary Table S5 Blood glucose during OGTT by group and time point.

Supplementary Table S6 Blood glucose during ITT by group and time.

## References

[1] Marie N Emmanuela G Justin L Yohannes HA Cristiana A Nasir A. Global, regional, and national prevalence of adult overweight and obesity, 1990-2021, with forecasts to 2050: a forecasting study for the Global Burden of Disease Study 2021. Lancet. (2025) 405:813–38. doi: 10.1016/S0140-6736(25)00355-1 40049186 PMC11920007

[2] Peri K Eisenberg M. Review on obesity management: diet, exercise and pharmacotherapy. BMJ Public Health. (2024) 2:e000246. doi: 10.1136/bmjph-2023-000246 41201084 PMC12581000

[3] Hajfathalian M Ghelichi S Jacobsen C. Anti-obesity peptides from food: production, evaluation, sources, and commercialization. Compr Rev Food Sci Food Saf. (2025) 24:e70158. doi: 10.1111/1541-4337.70158 40111015 PMC11924896

[4] Ali AH Li S Liu SQ Gan RY Li HB Kamal-Eldin A . Invited review: camel milk and gut health-Understanding digestibility and the effect on gut microbiota. J Dairy Sci. (2024) 107:2573–85. doi: 10.3168/jds.2023-23995 37977446

[5] Ali Redha A Valizadenia H Siddiqui SA Maqsood S A. state-of-art review on camel milk proteins as an emerging source of bioactive peptides with diverse nutraceutical properties. Food Chem. (2022) 373:131444. doi: 10.1016/j.foodchem.2021.131444 34717085

[6] Wang S Liu S He T Shen H Mo C Zhu Y . Camel-milk-derived extracellular vesicles mitigate high-fat-diet-induced obesity by enhancing thermogenesis and modulating lipid metabolism in mice. J Agric Food Chem. (2025) 73:21404–21. doi: 10.1021/acs.jafc.5c02577 40810347

[7] Hsu CL Schnabl B. The gut-liver axis and gut microbiota in health and liver disease. Nat Rev Microbiol. (2023) 21:719–33. doi: 10.1038/s41579-023-00904-3 37316582 PMC10794111

[8] Wang J Ren W Liu S Li Z Zeng Y Meng J . Changes in antioxidant capacity and gut microbiota in mice after intake of camel milk. Front Cell Infect Microbiol. (2025) 15:1621031. doi: 10.3389/fcimb.2025.1621031 40969552 PMC12440968

[9] Arab HH Eid AH Gad AM Yahia R Mahmoud AM Kabel AM. Inhibition of oxidative stress and apoptosis by camel milk mitigates cyclosporine-induced nephrotoxicity: targeting Nrf2/HO-1 and AKT/eNOS/NO pathways. Food Sci Nutr. (2021) 9:3177–90. doi: 10.1002/fsn3.2277 34136182 PMC8194908

[10] Zhang Y Wang J Ge W Song Y He R Wang Z . Camel milk peptides alleviate hyperglycemia by regulating gut microbiota and metabolites in type 2 diabetic mice. Food Res Int. (2023) 173:113278. doi: 10.1016/j.foodres.2023.113278 37803591

[11] Zheng Q Wang Y Chen G Mao X. Camel milk endogenous peptides ameliorated hyperglycemia in high-fat diet-fed C57BL/6 J mice in association with modulation of gut microbiota and the IRS/Akt and JNK/p38 pathways. Food Res Int. (2025) 212:116471. doi: 10.1016/j.foodres.2025.116471 40382047

[12] Han B Ye Y Zhang C Zhang L Zhou P A. Novel camel milk-derived peptide LLPK Improves glucose-lipid metabolism in db/db mice via PPAR signaling pathway. Nutrients. (2025) 17:1693. doi: 10.3390/nu17101693 40431433 PMC12113985

[13] Weng G Huang J Ma X Song M Yin Y Deng D . Brevibacillus laterosporus BL1, a promising probiotic, prevents obesity and modulates gut microbiota in mice fed a high-fat diet. Front Nutr. (2022) 9:1050025. doi: 10.3389/fnut.2022.1050025 36505236 PMC9729748

[14] Carrizales-Sanchez AK Tamez-Rivera O Garcia-Gamboa R Garcia-Cayuela T Rodriguez-Gutierrez NA Elizondo-Montemayor L . Gut microbial composition and functionality of school-age Mexican population with metabolic syndrome and type-2 diabetes mellitus using shotgun metagenomic sequencing. Front Pediatr. (2023) 11:1193832. doi: 10.3389/fped.2023.1193832 37342535 PMC10277889

[15] Liu T Lei C Huang Q Song W Li C Sun N . Hesperidin and fecal microbiota transplantation modulate the composition of the gut microbiota and reduce obesity in high fat diet mice. Diabetes Metab Syndr Obes. (2024) 17:3643–56. doi: 10.2147/DMSO.S474034 39398388 PMC11468570

[16] Santoso P Maliza R. Dietary fibers of Canna edulis and Maranta arundinacea rhizomes ameliorate metabolic diseases and gut dysbiosis in mice fed a high-fat diet. Res Pharm Sci. (2026) 21:47–63. doi: 10.4103/RPS.RPS_129_23 41607423 PMC12840878

[17] Herp S Durai Raj AC Salvado Silva M Woelfel S Stecher B. The human symbiont Mucispirillum schaedleri: causality in health and disease. Med Microbiol Immunol. (2021) 210:173–9. doi: 10.1007/s00430-021-00702-9 34021796 PMC7615636

[18] Wang Y Zhao L Huang S Peng J Wu K Wu M. High-fat diet-induced obesity disrupts the gut microbiome and mucus secretion to aggravate experimental colitis. Food Res Int. (2025) 221:117245. doi: 10.1016/j.foodres.2025.117245 41606850

[19] Gong J Shen Y Zhang H Cao M Guo M He J . Gut microbiota characteristics of people with obesity by meta-analysis of existing datasets. Nutrients. (2022) 14:2993. doi: 10.3390/nu14142993 35889949 PMC9325184

[20] Li Z Zhang L Wan Z Liu H Zhang T Li Y. Therapeutic potential of the gut commensal bacterium Parabacteroides goldsteinii in human health and disease treatment. Front Microbiol. (2025) 16:1618892. doi: 10.3389/fmicb.2025.1618892 41019532 PMC12461743

[21] Chen Y Jiang S Wang H Si M Wu H Liang X . The probiotic Parabacteroides Johnsonii Ameliorates metabolic disorders through promoting BCAAs to BSCFAs conversion. Adv Sci (Weinh). (2025) 12:e02624. doi: 10.1002/advs.202502624 40772426 PMC12520484

[22] Iqbal M Yu Q Tang J Xiang J. Unraveling the gut microbiota's role in obesity: key metabolites, microbial species, and therapeutic insights. J Bacteriol. (2025) 207:e0047924. doi: 10.1128/jb.00479-24 40183584 PMC12096833

[23] Ikeda T Takii K Omichi Y Nishimoto Y Ichikawa D Matsunaga T . Hexanoic acid improves metabolic health in mice fed high-fat Diet. Nutrients. (2025) 17:2868. doi: 10.3390/nu17172868 40944255 PMC12430857

[24] Green CD Maceyka M Cowart LA Spiegel S. Sphingolipids in metabolic disease: the good, the bad, and the unknown. Cell Metab. (2021) 33:1293–306. doi: 10.1016/j.cmet.2021.06.006 34233172 PMC8269961

[25] Juchnicka I Kuzmicki M Szamatowicz J. Ceramides and Sphingosino-1-Phosphate in obesity. Front Endocrinol (Lausanne). (2021) 12:635995. doi: 10.3389/fendo.2021.635995 34054722 PMC8158155

[26] Kajita K Ishii I Mori I Asano M Fuwa M Morita H. Sphingosine 1-Phosphate regulates obesity and glucose homeostasis. Int J Mol Sci. (2024) 25:932. doi: 10.3390/ijms25020932 38256005 PMC10816022

[27] Voss J Hornemann T Belgardt BF. Impact of nutrition on Sphingolipid-Regulated physiology: a review. Mol Nutr Food Res. (2025) 69:e70182. doi: 10.1002/mnfr.70182 40776813 PMC12538533

[28] Bai L Yang P Han B Kong L. Progress of the acyl-Coenzyme A thioester hydrolase family in cancer. Front Oncol. (2024) 14:1374094. doi: 10.3389/fonc.2024.1374094 38562172 PMC10982514

[29] Ma Y Jiang J Zhao C Wei B Gao J. Arachidonic acid metabolism in metabolic dysfunction-associated steatotic liver disease and liver fibrosis. Hepatol Commun. (2025) 9:802. doi: 10.1097/HC9.0000000000000802 40879475 PMC12401274

[30] Leahy C Osborne N Shirota L Rote P Lee YK Song BJ . The fatty acid omega hydroxylase genes (CYP4 family) in the progression of metabolic dysfunction-associated steatotic liver disease (MASLD): an RNA sequence database analysis and review. Biochem Pharmacol. (2024) 228:116241. doi: 10.1016/j.bcp.2024.116241 38697309 PMC11774579

[31] Li X Huang X Song K Liu J Jin Y Li T . Qingre Sanjie Formula alleviates atherosclerosis by promoting LXR-alpha/ABCG5/G8-mediated reverse cholesterol transport and bile acid synthesis. Phytomedicine. (2025) 142:156691. doi: 10.1016/j.phymed.2025.156691 40286749

[32] Zhang Q Guo WL Chen GM Qian M Han JZ Lv XC . Pediococcus acidilactici FZU106 alleviates high-fat diet-induced lipid metabolism disorder in association with the modulation of intestinal microbiota in hyperlipidemic rats. Curr Res Food Sci. (2022) 5:775–88. doi: 10.1016/j.crfs.2022.04.009 35520273 PMC9064835

[33] Liu T Zhang M Xie Q Gu J Zeng S Huang D. Unveiling the Antiobesity mechanism of sweet potato extract by microbiome, transcriptome, and metabolome analyses in mice. J Agric Food Chem. (2025) 73:7807–21. doi: 10.1021/acs.jafc.4c13173 39989409

[34] Ma L Wang H Jin Q Sun Z Yu S Zhang Y. The gut-liver axis: molecular mechanisms and therapeutic targeting in liver disease. Int J Gen Med. (2025) 18:7531–46. doi: 10.2147/IJGM.S551494 41424975 PMC12716074

[35] Ming L Qi B Hao S Ji R. Camel milk ameliorates inflammatory mechanisms in an alcohol-induced liver injury mouse model. Sci Rep. (2021) 11:22811. doi: 10.1038/s41598-021-02357-1 34819599 PMC8613211

[36] Ramos-Molina B Rossell J Perez-Montes de. Oca A, Pardina E, Genua I, Rojo-Lopez MI, et al. Therapeutic implications for sphingolipid metabolism in metabolic dysfunction-associated steatohepatitis. Front Endocrinol (Lausanne). (2024) 15:1400961. doi: 10.3389/fendo.2024.1400961 38962680 PMC11220194

[37] Bai H Xi G Cheng Y. Prostaglandin G/H synthase 1 promotes thrombosis in atrial fibrillation through modulation of platelet activation, macrophage infiltration, inflammation, and autophagy inhibition. Acta Cardiol. (2025) 80:254–65. doi: 10.1080/00015385.2025.2467009 39963884

[38] Che Z Xue W Zhao X Hu C Tian Y. Regulatory role and biomarker potential of gut microbiota metabolites in the progression of metabolic dysfunction-associated steatotic liver disease to hepatocellular carcinoma. Clin Transl Gastroenterol. (2025) 16:e00914. doi: 10.14309/ctg.0000000000000914 40911047 PMC12727350

[39] Yakut A. Gut microbiota in the development and progression of chronic liver diseases: gut microbiota-liver axis. World J Hepatol. (2025) 17:104167. doi: 10.4254/wjh.v17.i3.104167 40177197 PMC11959663

[40] Manaer T Yu L Nabi XH Dilidaxi D Liu L Sailike J. The beneficial effects of the composite probiotics from camel milk on glucose and lipid metabolism, liver and renal function and gut microbiota in db/db mice. BMC Complement Med Ther. (2021) 21:127. doi: 10.1186/s12906-021-03303-4 33888105 PMC8061000

[41] Ming L Qiao X Yi L Siren D He J Hai L . Camel milk modulates ethanol-induced changes in the gut microbiome and transcriptome in a mouse model of acute alcoholic liver disease. J Dairy Sci. (2020) 103:3937–49. doi: 10.3168/jds.2019-17247 32171514

[42] .Lee M, Min JH, Lee DE, Ryu JS, Hong SH, Hong Y, et al. Piperidinesulfonyl derivatives, novel CYP4A inhibitors, ameliorate hepatic lipotoxicity in metabolic dysfunction-associated steatotic liver disease. Biochem Pharmacol. (2026) 243, 117528. doi: 10.1016/j.bcp.2025.117528 41203031

[43] Pasini E Baciu C Angeli M Arendt B Pellegrina D Reimand J . Acyl-CoA Thioesterase 1 contributes to transition of steatosis to metabolic-associated steatohepatitis. Int J Hepatol. (2024) 2024:5560676. doi: 10.1155/2024/5560676 39624175 PMC11611406

[44] Yuan Q Zhang X Yang X Zhang Q Wei X Ding Z . Knockdown of ACOT4 alleviates gluconeogenesis and lipid accumulation in hepatocytes. Heliyon. (2024) 10:e27618. doi: 10.1016/j.heliyon.2024.e27618 38495177 PMC10940928

[45] Little M Ortlund EA. Structure, function, and lipid sensing activity in the thioesterase superfamily. Biochem Soc Trans. (2024) 52:1565–77. doi: 10.1042/BST20230313 39140379 PMC12004282

[46] Shu R Liu G Xu Y Liu B Huang Z Wang H. AcrAB efflux pump plays a crucial role in bile salts resistance and pathogenesis of Klebsiella pneumoniae. Antibiotics (Basel). (2024) 13:1146. doi: 10.3390/antibiotics13121146 39766536 PMC11672700

[47] Aljutaily T Aladhadh M Alsaleem KA Alharbi HF Barakat H Aljumayi H . Gut microbiota diversity in obese rats treated with intermittent fasting, probiotic-fermented camel milk with or without dates and their combinations. Sci Rep. (2025) 15:14204. doi: 10.1038/s41598-025-96893-9 40269059 PMC12019252

[48] Seyiti S Kelimu A Yusufu G. Bactrian camel milk: chemical composition, bioactivities, processing techniques, and economic potential in China. Molecules. (2024) 29:4680. doi: 10.3390/molecules29194680 39407609 PMC11478162

