# Supplementary material for: Camel milk is associated with ameliorated high-fat diet-induced obesity and metabolic disorders via modulation of gut microbiota and remodeling of hepatic lipid metabolism: a multi-omics analysis
Source: Front Nutr. 2026 Oct 22;13:1913436. doi: 10.3389/fnut.2026.1913436 (PMC13736606; doi:10.3389/fnut.2026.1913436)
Supplement: Supplementary file 1 [file Supplementary_file_1.docx]

**Detailed methods for SCFA analysis**

**Derivatization reagent and the 150 μL volume**

The 150 μL of derivatization reagent a mixture of three components: 50 μL of 160 mM 3-NPH-d4 (3-nitrophenylhydrazine-d4) in 80% methanol/water, 50 μL of 120 mM EDC (N-(3-dimethylaminopropyl)-N′-ethylcarbodiimide) in methanol, and 50 μL of 8% pyridine in methanol.
**Internal standards**
The mixed internal standard solution (5 μL added to 95 μL of supernatant) contained the following deuterated SCFAs: Acetic acid-D4, Propionic acid-D4, Butyric acid-D4, Isobutyric acid-D4, Valeric acid-D4, Isovaleric acid-D4, 2-Methylbutyrate-D4, Hexanoic acid-D4, 4-Methylvaleric acid-D4, 2-Methylvalerate-D4, and 3-Methylvalerate-D4.

**Calibration curve linearity**

Calibration curves were constructed for each SCFA by plotting the peak area ratio (analyte/internal standard) against the standard concentration. All curves showed excellent linearity (R² > 0.99). The detailed linearity ranges for each compound are provided in Supplementary Table S2.

**Complete chromatographic and mass spectrometric conditions.**

The full UHPLC-MS/MS conditions have been included in the Supplementary Methods. Briefly, separation was performed on a Waters ACQUITY UPLC BEH C18 column (2.1 × 100 mm, 1.7 μm) at 40 °C, using a gradient of 10 mM ammonium acetate in water (A) and acetonitrile/isopropanol (1:1) (B) at 0.30 mL/min. The mobile phase, consisting of 10 mM ammonium acetate in water (solvent A) and acetonitrile: isopropanol (1:1) (solvent B), was delivered at a flow rate of 0.30 mL/min. The solvent gradient was set as follows: initial 25% B, 2.5min; 25-30% B, 3 min; 30-35% B, 3.5 min; 35-38% B, 4 min; 38-40% B, 4.5 min; 40-45% B, 5 min; 45-50% B, 5.5min; 50-55% B, 6.5 min; 55-58% B, 7 min; 58-70% B, 7.5min; 70-100% B, 7.8 min; 100-25% B, 10.1min; 25% B, 12 min. The mass spectrometer was operated in negative MRM mode with IonSpray Voltage −4500 V, Sheath Gas 35 psi, Ion Source Temperature 550 °C, Auxiliary Gas 50 psi, and Collision Gas 55 psi.

**Data processing, integration, and normalization**

Quantification was performed using TraceFinder software. For each analyte, the peak area was integrated, and the ratio to the corresponding internal standard was calculated. Concentrations in samples were determined from the calibration curves. No further normalization was applied.

**Detailed methods for untargeted liver metabolomics**

Metabolites were extracted from 100 mg of liver tissue using 500 µL of 80% aqueous methanol. After incubation on ice for 5 min, the samples were centrifuged at 15,000 × g and 4 °C for 20 min. A volume of 120 µL of the supernatant was diluted with water to a final methanol concentration of 53%, followed by another centrifugation step under the same conditions (15,000 × g, 20 min, 4 °C). The resulting supernatant was collected and analyzed by LC–MS.

**Chromatographic parameters**
Column temperature: 40 °C.

Injection volume: 5 µL.

Flow rate: 0.2 mL/min.

Gradient conditions:2% B, 1.5 min; 2-100% B, 7 min; 100% B, 9 min；100-2% B, 9.1 min；2% B, 12 min.

Eluent A: 5 mM ammonium acetate in 90% acetonitrile; eluent B: 5 mM ammonium acetate in 50% acetonitrile (used for both positive and negative polarity modes).

**Mass spectrometry parameters**
All relevant MS settings are now fully listed:

Capillary/cone voltage: 3.2 kV (ESI+), 2.4 kV (ESI−).

Source temperature: 120 °C.

Desolvation temperature: 350 °C.

Cone gas flow: 25 L h⁻¹.

Desolvation gas flow: 900 L h⁻¹.

Additional Orbitrap-specific parameters: spray voltage 3.5 kV, capillary temperature 320 °C, sheath gas 35 arb, auxiliary gas 10 arb, S-lens RF level 60, auxiliary gas heater temperature 350 °C

**Data processing, normalization and metabolite identification**
Raw UHPLC–MS/MS data files were processed using XCMS for peak alignment, peak picking and quantitation. Metabolites were identified by matching accurate mass and MS/MS spectra to the self-built high-quality NovoMetDB database (mass deviation ≤ 10 ppm), with adduct ion information taken into account. Background ions were removed based on blank samples. Normalization was performed using the formula: Relative peak area = raw quantitative value of the sample / (sum of quantitative values of all samples/sum of quantitative values of QC1). Compounds with a coefficient of variation (CV) of relative peak areas in QC samples > 30% were filtered out. All data processing was carried out on CentOS 6.6 using R and Python; detailed package and software versions are documented in the README file accompanying the results.
