# Supplementary material for: Camel milk is associated with ameliorated high-fat diet-induced obesity and metabolic disorders via modulation of gut microbiota and remodeling of hepatic lipid metabolism: a multi-omics analysis
Source: Front Nutr. 2026 Oct 22;13:1913436. doi: 10.3389/fnut.2026.1913436 (PMC13736606; doi:10.3389/fnut.2026.1913436)
Supplement: Supplementary file 2 [file Table_1.docx]

Supplementary Table S1 Camel milk composition.

| Fat (%) | Protein (%) | Lactose (%) | Total solids (%) |
| --- | --- | --- | --- |
| 4.57 | 3.62 | 5.28 | 14.50 |
