# Supplementary material for: Camel milk is associated with ameliorated high-fat diet-induced obesity and metabolic disorders via modulation of gut microbiota and remodeling of hepatic lipid metabolism: a multi-omics analysis
Source: Front Nutr. 2026 Oct 22;13:1913436. doi: 10.3389/fnut.2026.1913436 (PMC13736606; doi:10.3389/fnut.2026.1913436)
Supplement: Supplementary file 3 [file Table_3.docx]

Supplementary Table S3 Analysis of BW measurements in each group.

| Week | NC | HFD | Orlistat | CM |
| --- | --- | --- | --- | --- |
| 10 | 26.15±1.68^b^ | 34.85±2.27^a^ | 33.51±1.55^a^ | 32.94±2.22^a^ |
| 11 | 26.88±1.71^b^ | 35.64±2.64^a^ | 32.79±1.67^a^ | 33.28±2.18^a^ |
| 12 | 26.69±1.82^b^ | 35.88±2.63^a^ | 32.83±2.23^a^ | 33.34±1.30^a^ |
| 13 | 26.13±1.98^c^ | 36.63±2.90^a^ | 33.06±2.89^ab^ | 32.00±1.65^b^ |
| 14 | 24.98±2.02^c^ | 35.38±3.09^a^ | 32.44±2.74^ab^ | 29.84±2.78^b^ |
| 15 | 21.24±1.37^c^ | 34.65±2.76^a^ | 29.93±3.79^ab^ | 26.36±2.61^b^ |
| 16 | 20.78±1.84^c^ | 32.54±4.35^a^ | 27.96±4.46^ab^ | 25.5±3.03^b^ |
| 17 | 19.68±1.5^b^ | 29.66±4.19^a^ | 26.94±4.80^a^ | 26.04±2.76^a^ |

Bars with different lowercase letters (a, b, c) indicate statistically significant differences between groups (*p* < 0.05, two-way repeated-measures ANOVA followed by Tukey’s post hoc test). Groups sharing a common letter are not significantly different.

Supplementary Table S4 Food intake in each group.

| Week | NC | HFD | Orlistat | CM |
| --- | --- | --- | --- | --- |
| 11 | 2.600±0.18^a^ | 2.175±0.15^b^ | 2.150±0.19^ab^ | 2.225±0.12^ab^ |
| 12 | 2.600±0.25^a^ | 2.650±0.40^a^ | 2.400±0.25^a^ | 2.325±0.29^a^ |
| 13 | 2.425±0.17^a^ | 2.375±0.17^a^ | 2.575±0.22^a^ | 1.900±0.48^a^ |
| 14 | 1.950±0.19^bc^ | 2.200±0.14^ab^ | 2.500±0.16^a^ | 1.775±0.17^c^ |
| 15 | 1.775±0.47^ab^ | 2.050±0.31^a^ | 2.100±0.40^a^ | 1.000±0.11^b^ |
| 16 | 1.950±0.25^a^ | 1.700±0.71^a^ | 2.075±0.83^a^ | 1.750±0.47^a^ |
| 17 | 1.900±0.28^a^ | 1.250±0.23^b^ | 2.000±0.25^a^ | 1.625±0.56^ab^ |

Bars with different lowercase letters (a, b, c) indicate statistically significant differences between groups (*p* < 0.05, two-way repeated-measures ANOVA followed by Tukey’s post hoc test). Groups sharing a common letter are not significantly different.

Supplementary Table S5 Blood glucose during OGTT by group and time point.

| Time（min） | NC | HFD | Orlistat | CM |
| --- | --- | --- | --- | --- |
| 0 | 4.500±0.64^b^ | 7.363±1.14^a^ | 4.663±0.36^b^ | 5.225±1.63^b^ |
| 30 | 12.15±1.39^c^ | 19.84±1.77^a^ | 11.74±1.58^c^ | 15.68±1.56^b^ |
| 60 | 8.000±1.51^b^ | 15.88±2.70^a^ | 8.663±1.21^b^ | 10.78±2.25^b^ |
| 90 | 6.838±1.78^b^ | 12.09±1.52^a^ | 7.525±1.30^b^ | 9.138±2.44^ab^ |
| 120 | 6.563±1.30^b^ | 10.78±1.35^a^ | 7.238±1.19^b^ | 8.813±2.49^ab^ |

Bars with different lowercase letters (a, b, c) indicate statistically significant differences between groups (*p* < 0.05, two-way repeated-measures ANOVA followed by Tukey’s post hoc test). Groups sharing a common letter are not significantly different.

Supplementary Table S6 Blood glucose during ITT by group and time.

| Time（min） | NC | HFD | Orlistat | CM |
| --- | --- | --- | --- | --- |
| 0 | 7.300±1.60^ab^ | 8.750±0.93^a^ | 5.900±1.24^b^ | 7.263±1.37^ab^ |
| 30 | 4.675±1.37^ab^ | 5.113±0.73^a^ | 3.925±0.54^b^ | 4.600±1.44^ab^ |
| 60 | 5.000±1.90^ab^ | 4.700±0.55^a^ | 3.225±0.38^b^ | 4.538±1.65^ab^ |
| 90 | 5.963±1.85^a^ | 5.675±1.54^a^ | 3.375±1.72^b^ | 4.800±2.45^ab^ |
| 120 | 6.925±1.95^a^ | 7.100±1.86^a^ | 4.700±1.52^a^ | 6.213±2.20^a^ |

Bars with different lowercase letters (a, b, c) indicate statistically significant differences between groups (*p* < 0.05, two-way repeated-measures ANOVA followed by Tukey’s post hoc test). Groups sharing a common letter are not significantly different.
